# Supplementary figures and images for: Anthracyclines induce global changes in cardiomyocyte chromatin accessibility that overlap with cardiovascular disease loci
Source: PLoS Genet. 2025 Oct 14;21(10):e1011900. doi: 10.1371/journal.pgen.1011900 (PMC12561927; doi:10.1371/journal.pgen.1011900)

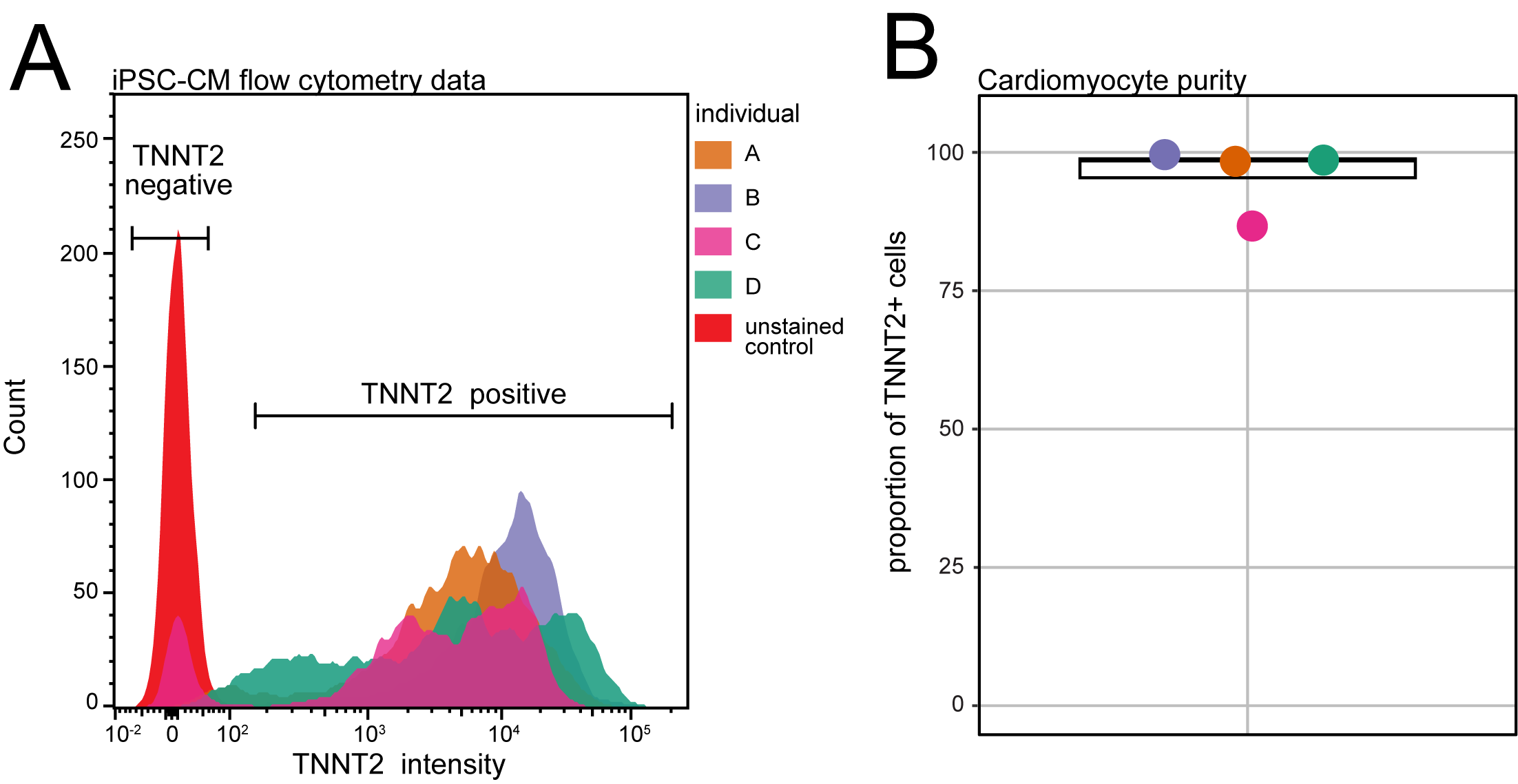

Supplement: S1 Fig — (A) Flow cytometry results indicating the proportion of TNNT2-positive cells following differentiation in Individuals A, B, C, D as well as unlabeled iPSC-CMs from a mixture of Individuals A and D. (B) Proportion of TNNT2-positive cells from each individual (A: orange; B: purple; C: magenta; D: teal). Data from each individual is presented as the mean of two replicate flow cytometry experiments. (TIF) [file pgen.1011900.s025.tif]

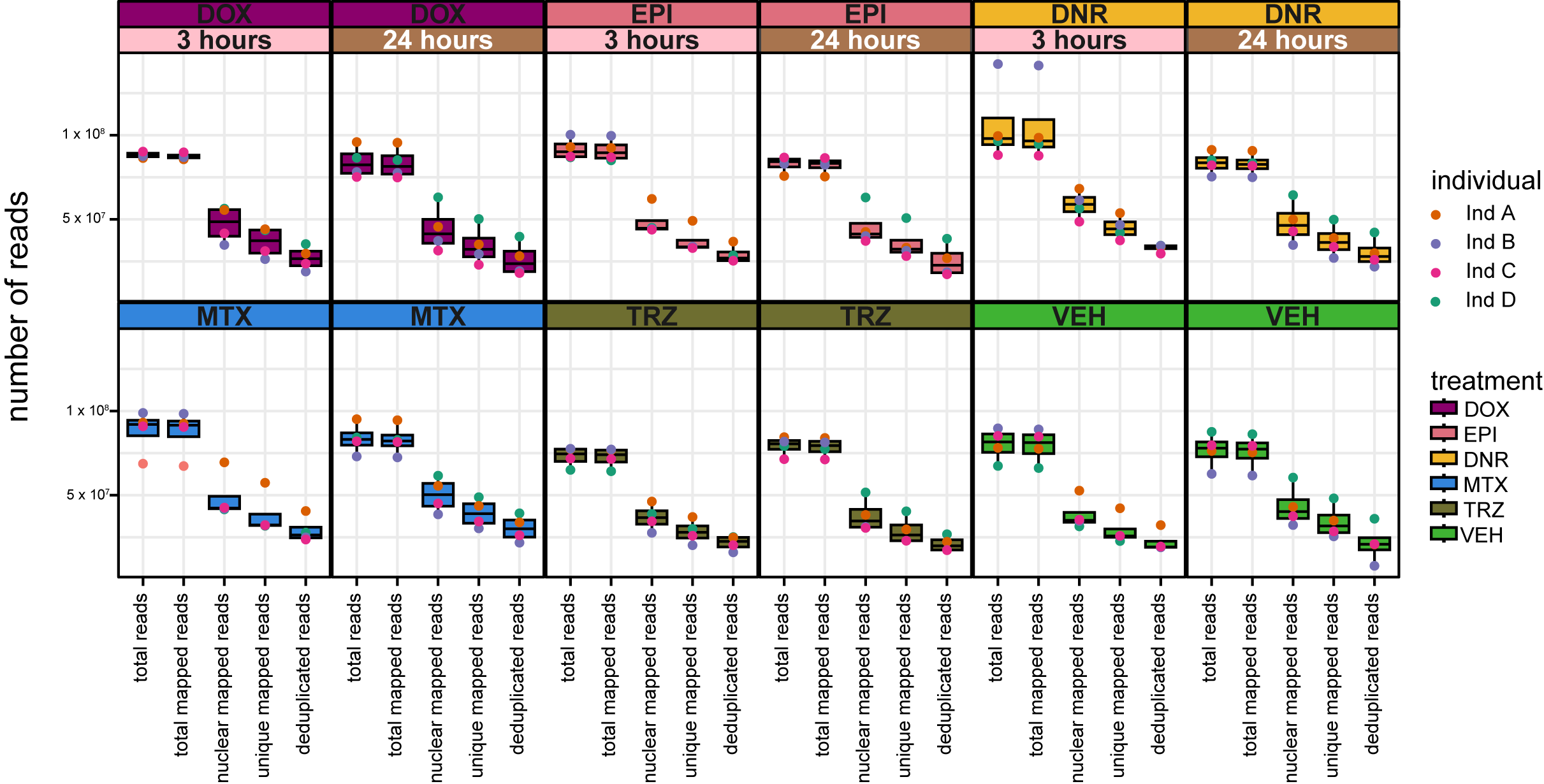

Supplement: S2 Fig — The number of total reads, total mapped reads, total nuclear-mapped reads, unique-nuclear mapped reads, and deduplicated-unique-nuclear mapped reads by time and treatment for four individuals. Samples for each individual (A: orange; B: purple; C: magenta; D: teal) are grouped by treatment and time. Read numbers represent the sum of read 1 and read 2 for each sample. (TIF) [file pgen.1011900.s026.tif]

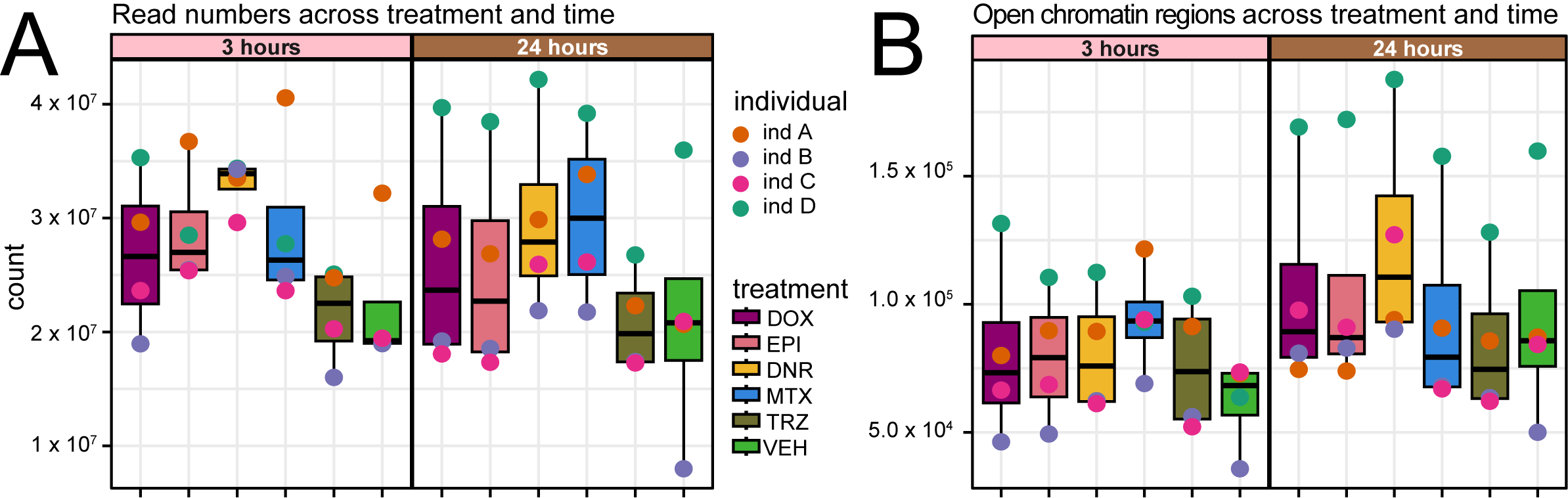

Supplement: S3 Fig — (A) Number of unique-deduplicated reads (read 1 + read 2 per sample) across time and treatment (DOX: mauve; EPI: pink; DNR: yellow; MTX: blue; TRZ: olive; VEH: green) at three and 24 hours for each individual (A: orange; B: purple; C: magenta; D: teal). (B) Number of open chromatin regions called per treatment and time for each individual. (TIF) [file pgen.1011900.s027.tif]

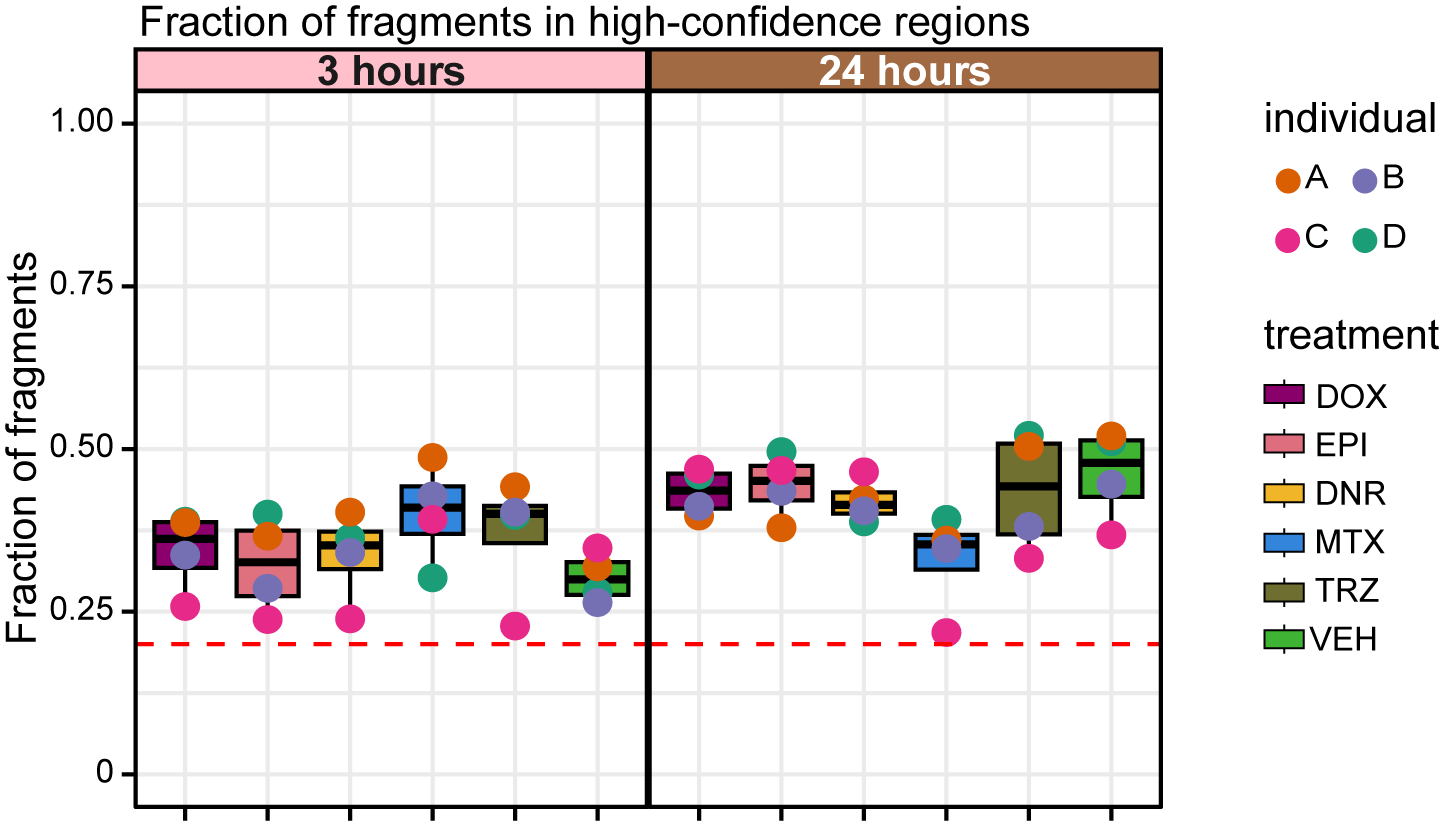

Supplement: S4 Fig — The fraction of fragments in a set of high-confidence open chromatin regions (n = 172,481) for each individual (A: orange; B: purple; C: magenta; D: teal) across time and treatment (DOX: mauve; EPI: pink; DNR: yellow; MTX: blue; TRZ: olive; VEH: green). All samples meet or exceed the minimum acceptable quality metric of 0.2 used by ENCODE (dashed red line). (TIF) [file pgen.1011900.s028.tif]

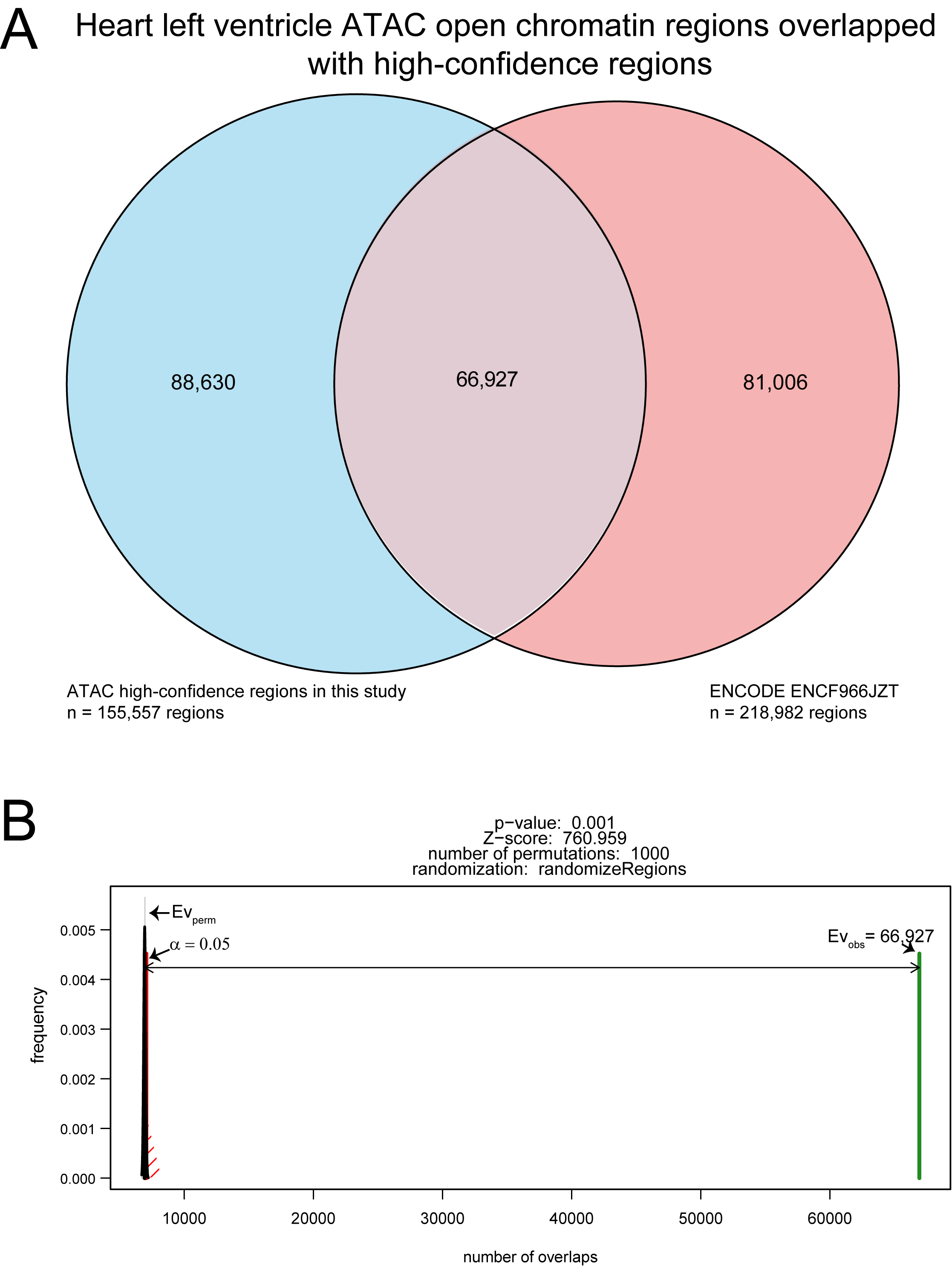

Supplement: S5 Fig — (A) Overlap between our 155,557 filtered high-confidence open chromatin regions in iPSC-CMs and open chromatin regions from heart left ventricle tissue from a 41-year-old female (ATAC-seq file ENCF966JZT from ENCODE experiment ENCSR204PZT). (B) Permutation test to determine significance of the overlap. (TIF) [file pgen.1011900.s029.tif]

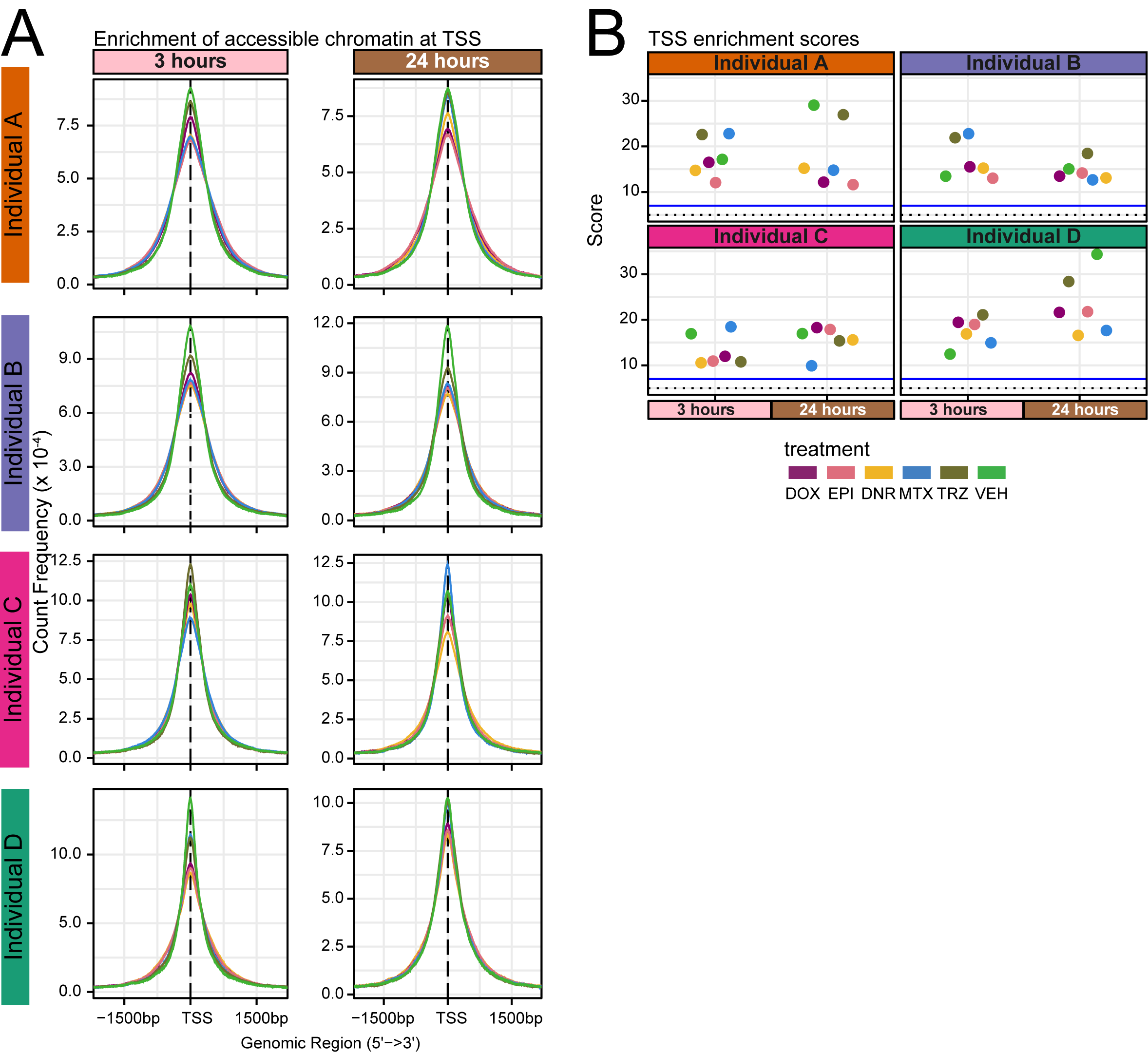

Supplement: S6 Fig — (A) Count frequency of fragments that map within +/-1.5 kb of all transcription start sites (TSS) across samples by individual and time. The dashed line is the TSS location. Solid lines are colored by treatment (DOX: mauve; EPI: pink; DNR: yellow; MTX: blue; TRZ: olive; VEH: green). (B) TSS enrichment scores across individuals by treatment and time. Dots represent treatment for the individuals listed above. The ENCODE ATAC-seq TSS enrichment score thresholds for the minimum ideal (solid blue line) and minimum acceptable threshold (dotted black line) are also shown. (TIF) [file pgen.1011900.s030.tif]

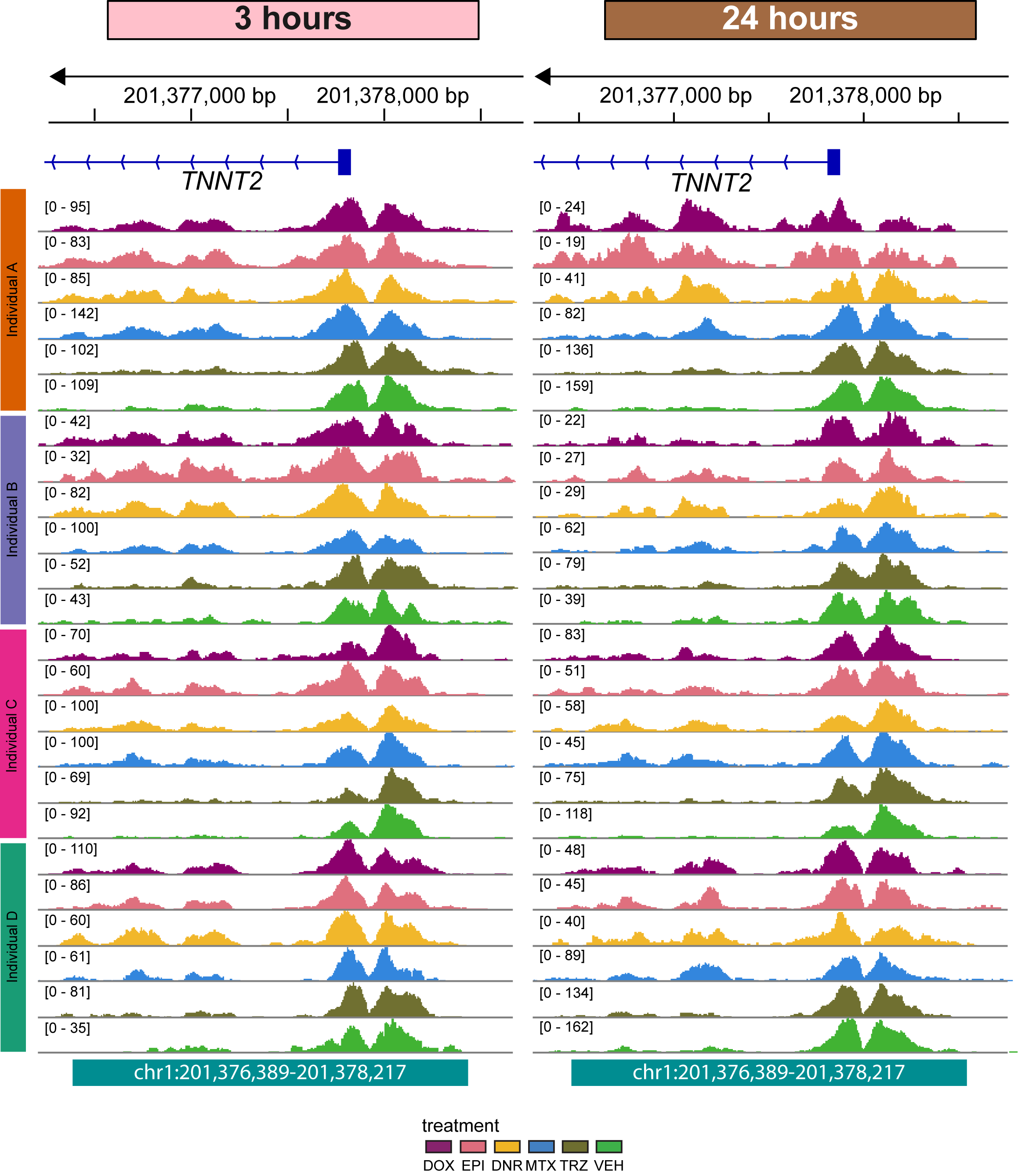

Supplement: S7 Fig — ATAC-seq fragments at a representative open chromatin region (chr1:201,376,390–201,378,217) at the TSS of troponin T (TNNT2) for all samples across time and drug treatments. Three-hour samples are shown on the left, 24-hour samples are on the right. Samples are grouped by individual and colored by treatment (DOX: mauve; EPI: pink; DNR: yellow; MTX: blue; TRZ: olive; VEH: green). (TIF) [file pgen.1011900.s031.tif]

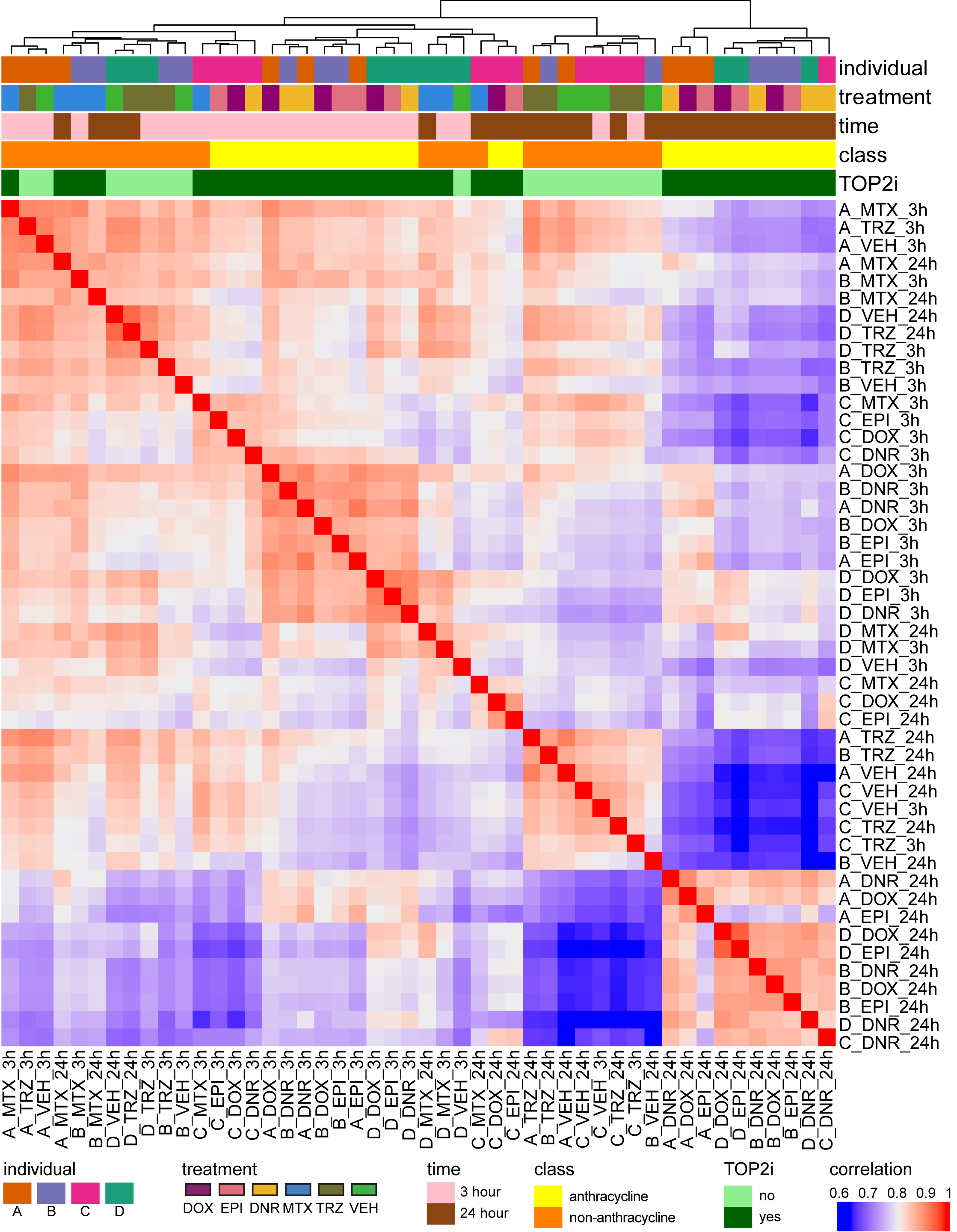

Supplement: S8 Fig — Pearson correlation of log2 cpm values across all pairs of samples for the high-confidence set of 155,557 open chromatin regions. Top bars are colored by individual (A: orange; B: purple; C: magenta; D: teal), treatment (DOX: mauve; EPI: pink; DNR: yellow; MTX: blue; TRZ: olive; VEH: green), time (three hours: pink; 24 hours: brown), class (anthracycline: yellow; non-anthracycline: orange), and classification as a TOP2i (no: light green; yes: dark green). (TIF) [file pgen.1011900.s032.tif]

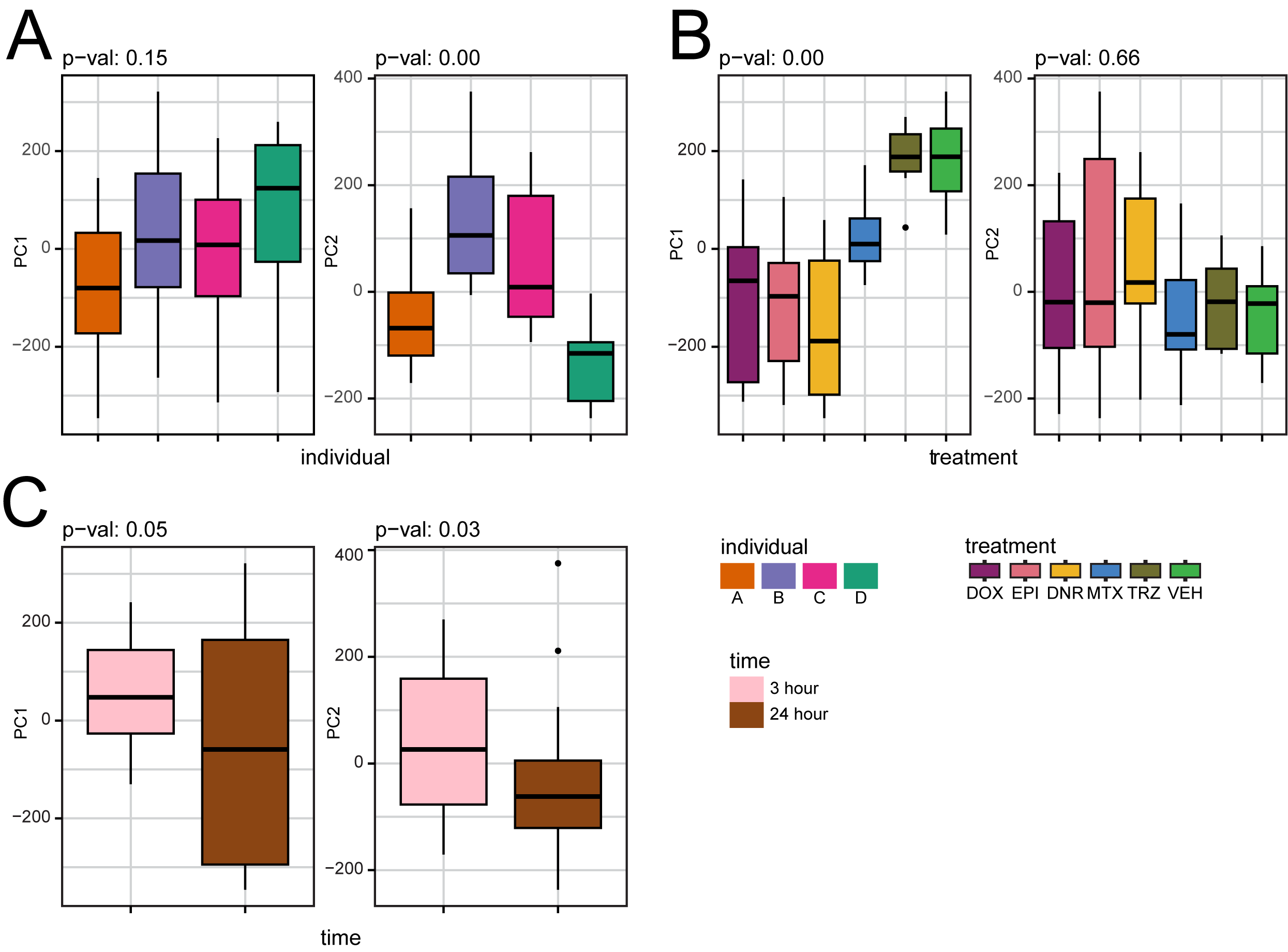

Supplement: S9 Fig — Demonstration of variance contributed to the first two principal components (PC) by individual, treatment, and time (the three major biological factors in this study). (A) Variance of individual (A: orange; B: purple, C: magenta; D: teal) as a function of PC1 and PC2. The correlation between individual and each PC is calculated using a linear model. P values represent the significance of the F-statistic from the model. (B) Variance of treatment (DOX: mauve; EPI: pink; DNR: yellow; MTX: blue; TRZ: olive; VEH: green) as a function of PC1 and PC2. (C) Variance of time (three hour: pink; 24 hour: brown) as a function of PC1 and PC2. (TIF) [file pgen.1011900.s033.tif]

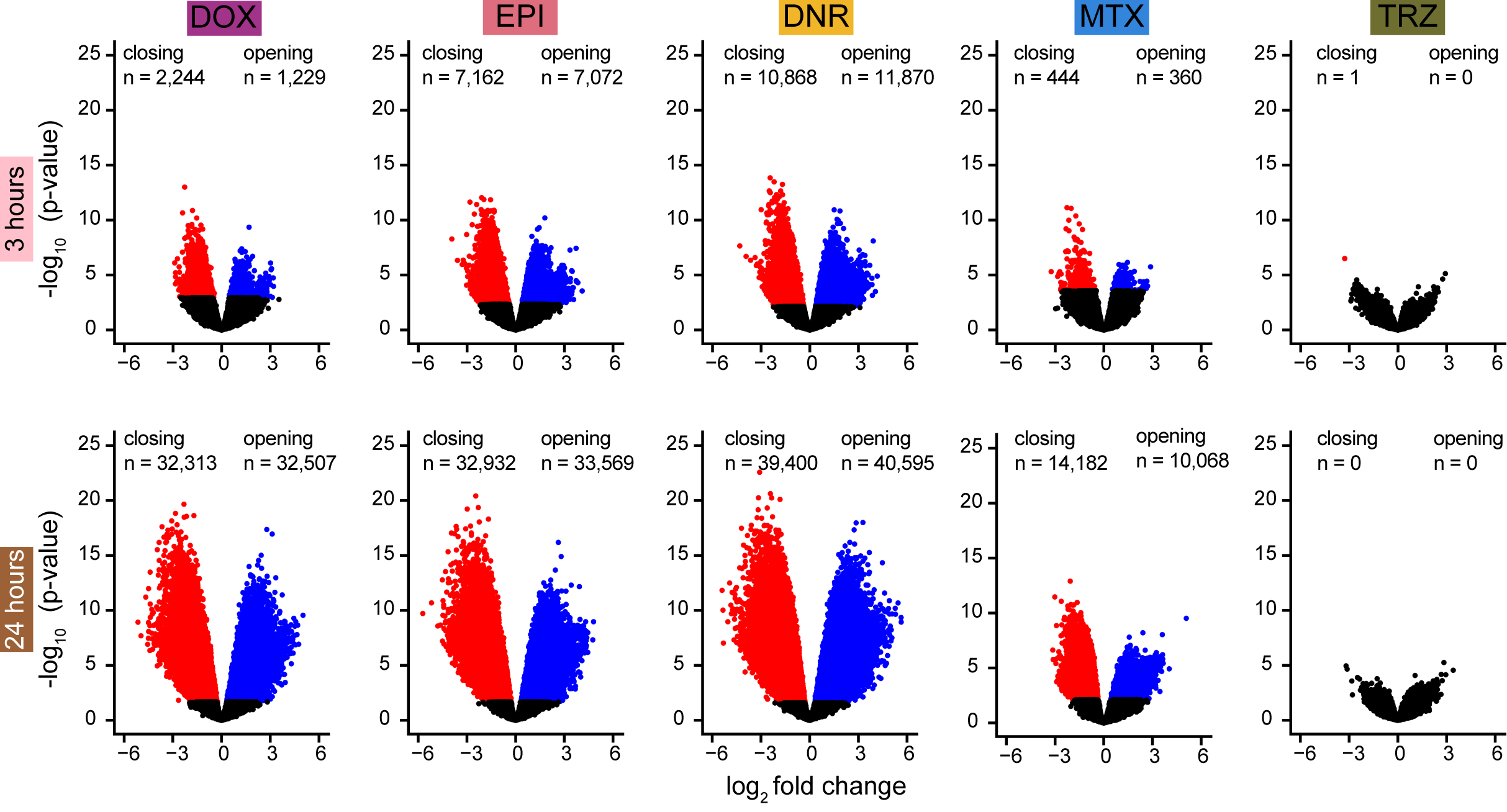

Supplement: S10 Fig — Volcano plots representing open chromatin regions that are differentially accessible (adjusted P < 0.05) in each drug treatment compared to VEH at each timepoint. Regions that increase in accessibility in response to treatment (opening) are represented in blue, and regions that significantly decrease in accessibility (closing) are represented in red. (TIF) [file pgen.1011900.s034.tif]

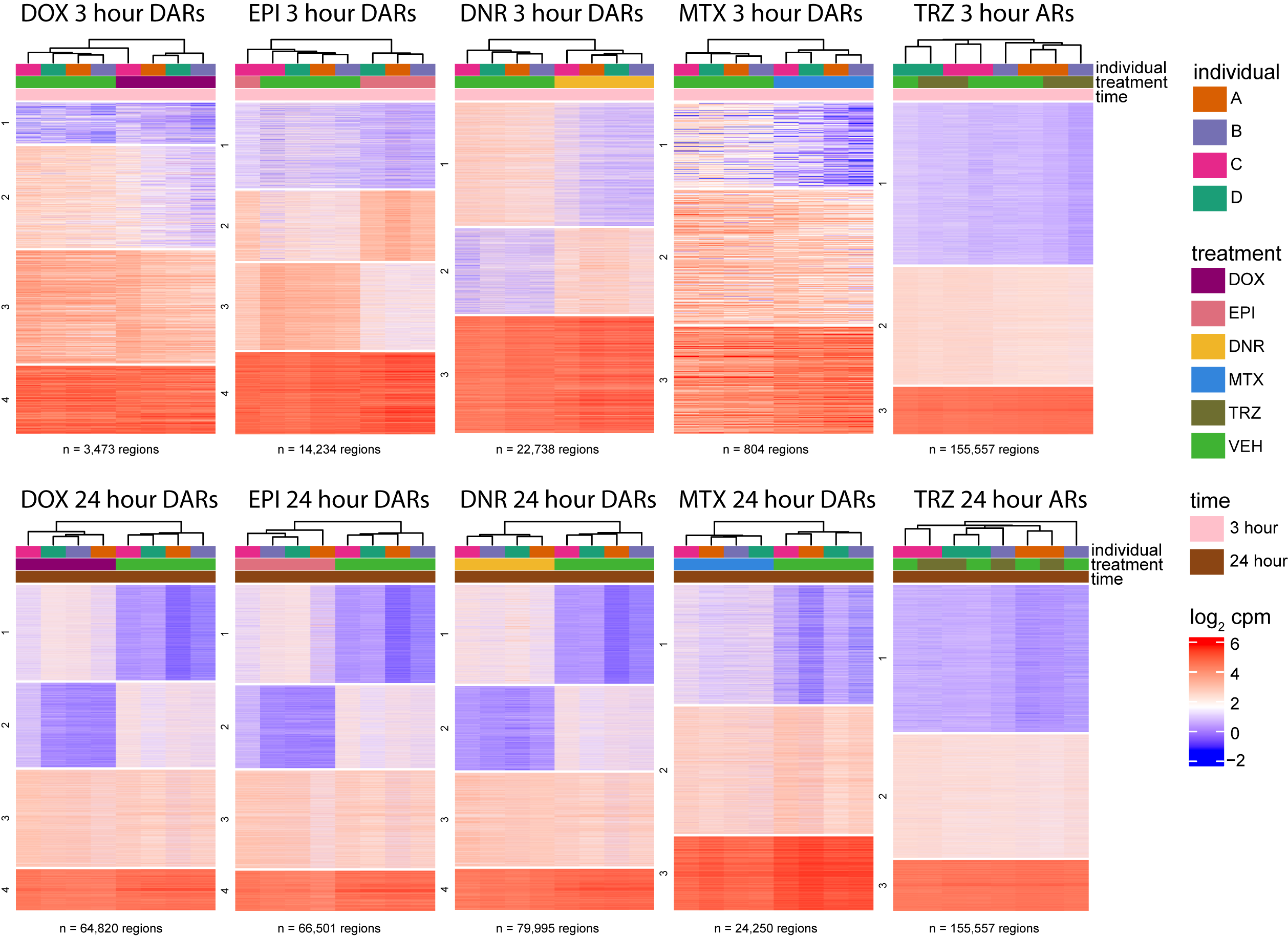

Supplement: S11 Fig — Chromatin accessibility (log2 cpm) at regions classified as DARs across treatments. Given that there is only one DAR in response to TRZ treatment, all accessible regions (ARs) are represented for this treatment. A common color scale was applied to all heatmaps to allow consistent comparisons across conditions. The scale was based on the global distribution of log2 cpm values across all samples. To minimize the influence of outlier expression values, the color scale was capped at the first and 99th percentiles of the global log2 cpm distribution, mapping the lowest 1% to blue and the highest 1% to red. The median log2 cpm value was set to white. (TIF) [file pgen.1011900.s035.tif]

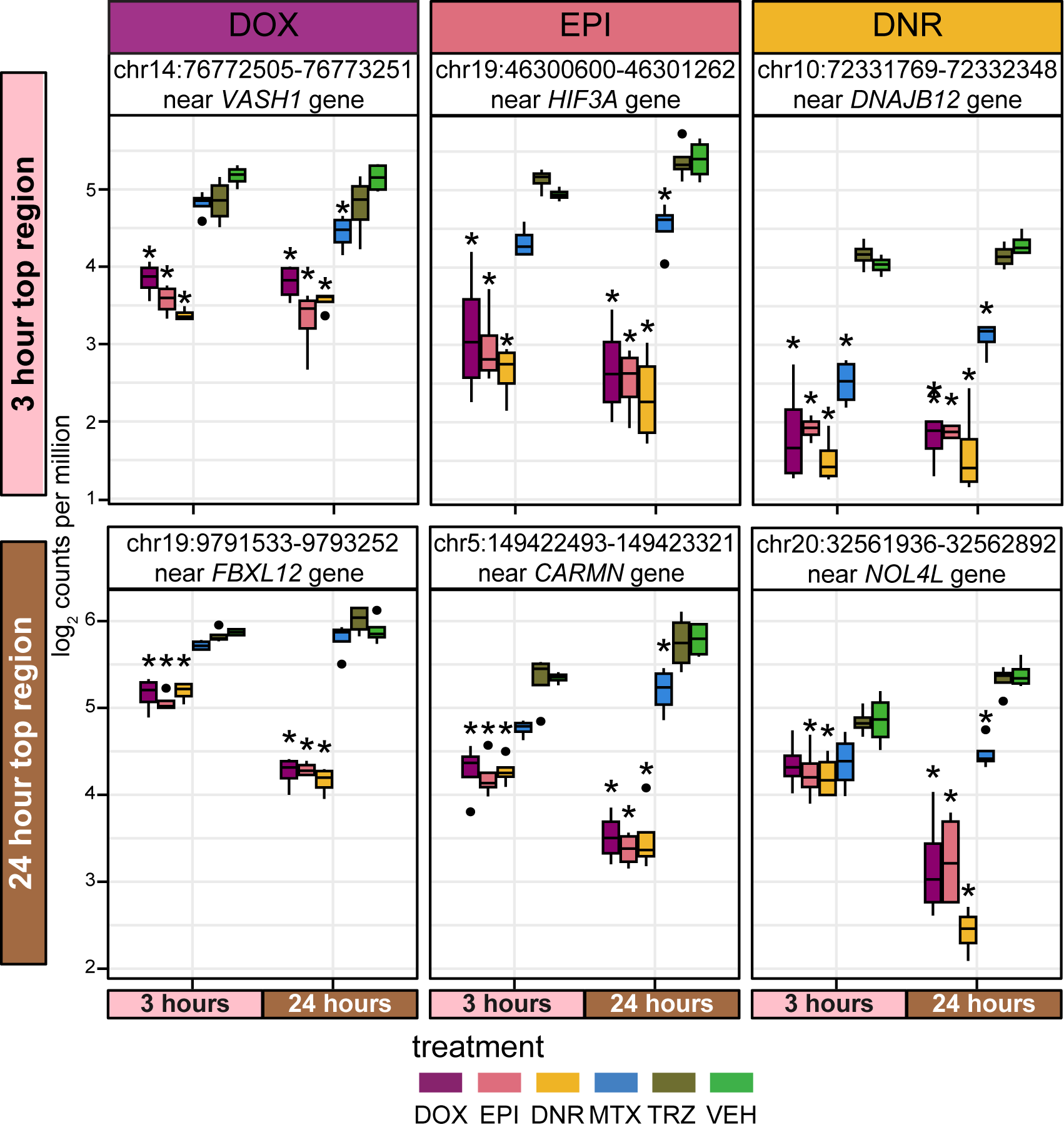

Supplement: S12 Fig — (A) Chromatin accessibility (log2 cpm) at top differentially accessible regions for DOX, EPI, and DNR at three and 24 hours across each treatment (DOX: mauve; EPI: pink; DNR: yellow; MTX: blue; TRZ: olive; VEH: green). Asterisk represents regions classified as a DAR following each treatment (adjusted P < 0.05). (TIF) [file pgen.1011900.s036.tif]

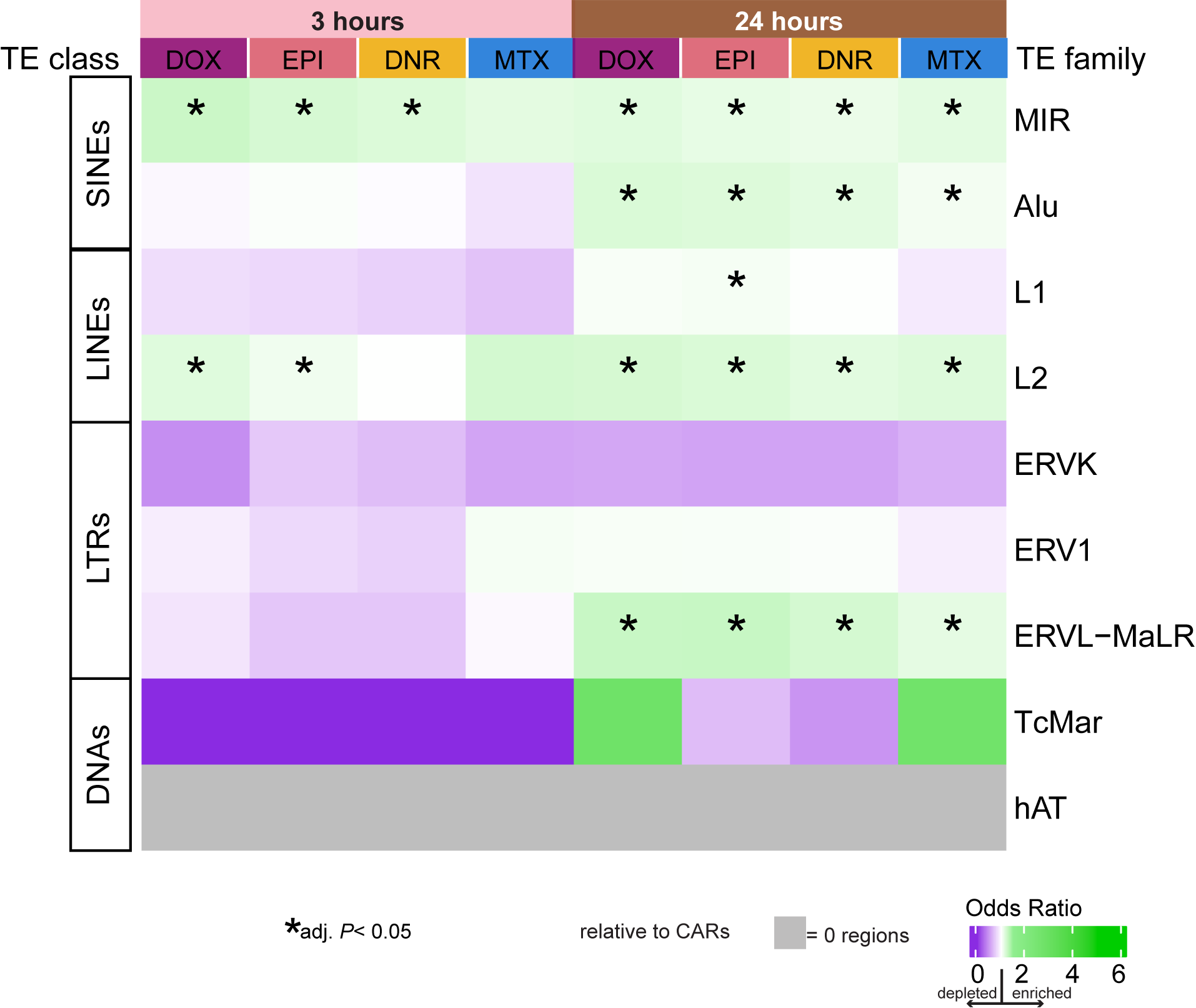

Supplement: S13 Fig — Enrichment of TE families in DARs compared to CARs for each drug treatment determined by Chi-square test and Benjamini-Hochberg multiple testing correction. Asterisk represents TE families that are significantly enriched. (TIF) [file pgen.1011900.s037.tif]

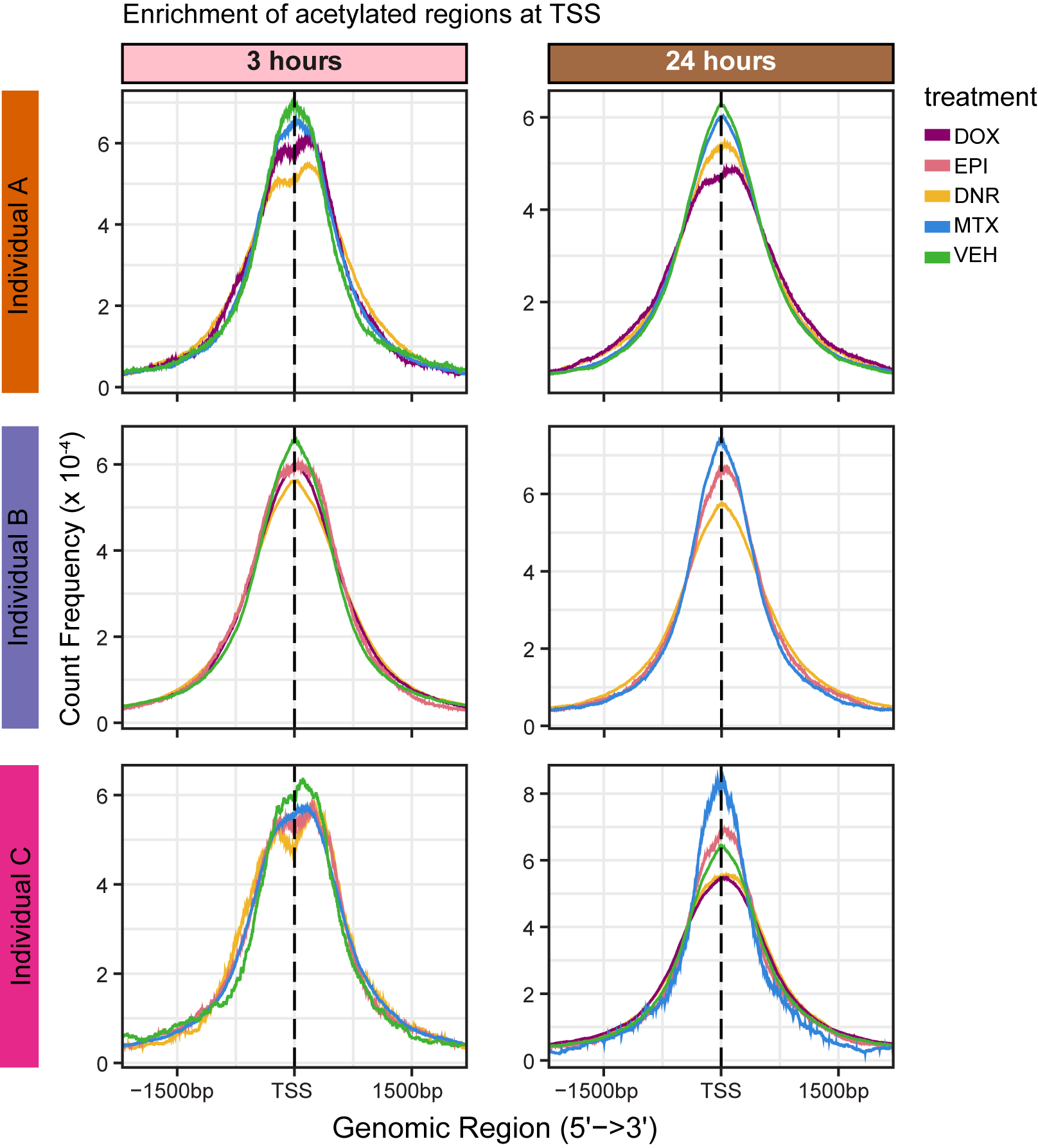

Supplement: S14 Fig — Count frequency of H3K27ac regions that map within +/-1.5 kb of all TSS in the hg38 genome across samples by individual and time. The dashed line is the TSS location. Solid lines are colored by treatment (DOX: mauve; EPI: pink; DNR: yellow; MTX: blue; VEH: light green). Individual plots with fewer than five solid lines indicate that those library preparations failed. (TIF) [file pgen.1011900.s038.tif]

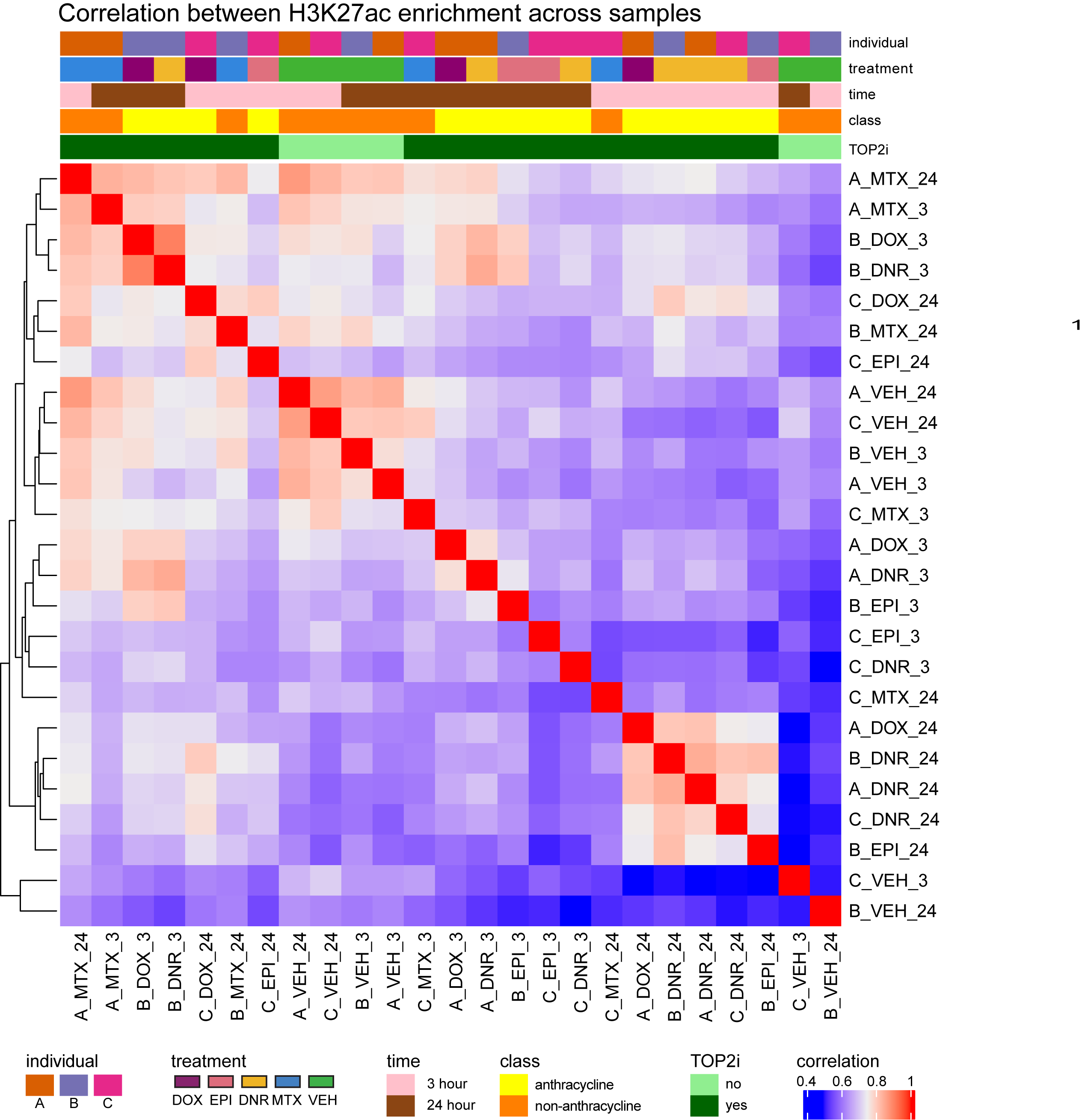

Supplement: S15 Fig — Pearson correlation of log2 cpm values across 20,137 high-confidence H3K27ac-enriched regions. Colored bars represent individual (A: orange; B: purple; C: magenta; D: teal), treatment (DOX: mauve; EPI: pink; DNR: yellow; MTX: blue; TRZ: olive; VEH: green), time (three hours: pink; 24 hours: brown), class (anthracycline: yellow; non-anthracycline: orange), and classification as a TOP2i (no: light green; yes: dark green). (TIF) [file pgen.1011900.s039.tif]

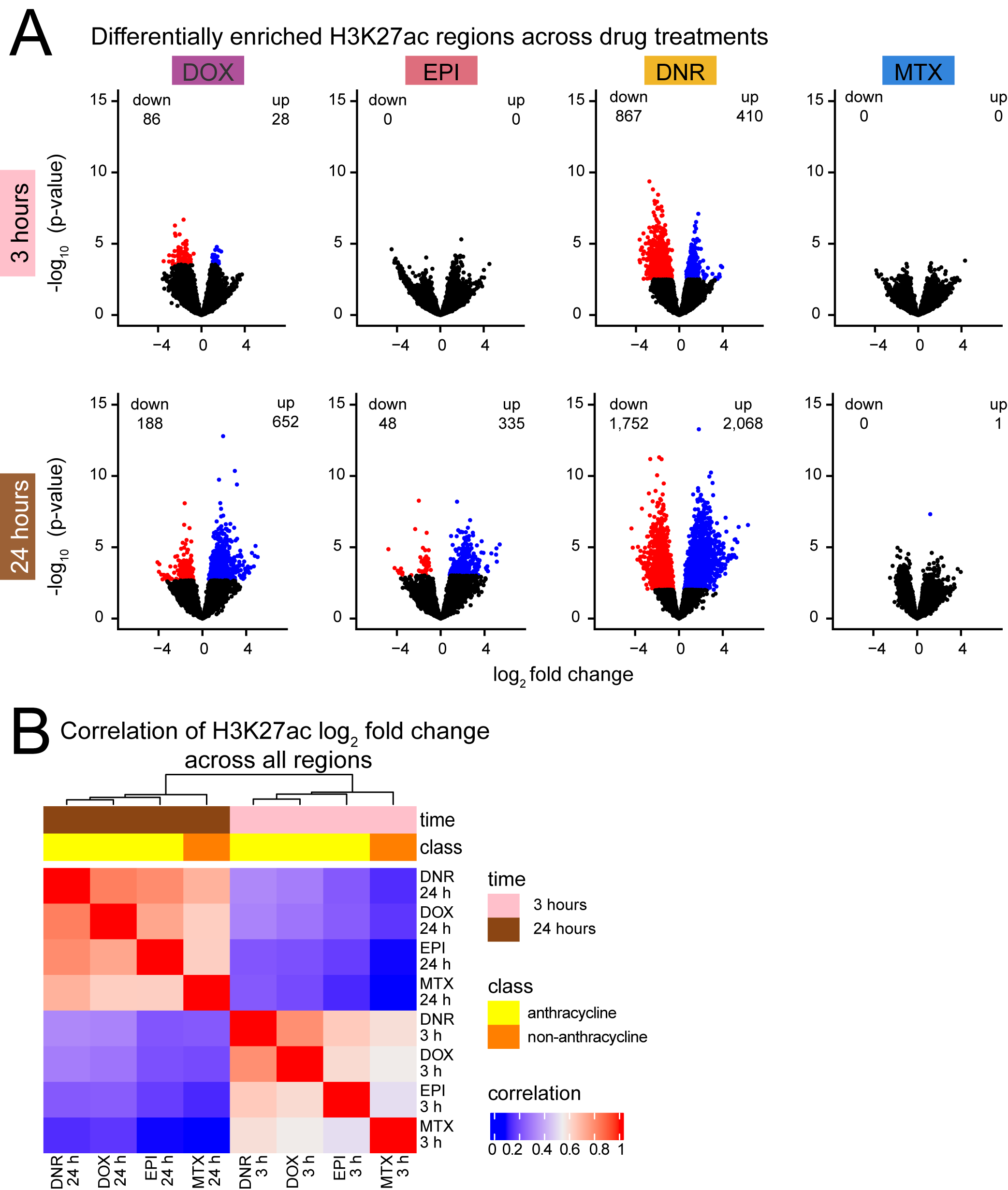

Supplement: S16 Fig — (A) Volcano plots representing H3K27ac enrichment changes for each drug compared to VEH at each timepoint across treatments (adjusted P < 0.05). Regions that increase in acetylation in response to treatment are represented in blue (up), and regions that decrease in acetylation in response to treatment are represented in red (down). (B) Pearson correlation of H3K27ac drug response across time and treatment. Colored bars represent time (three hours: pink; 24 hours: brown), and class (anthracycline: yellow; non-anthracycline: orange). (TIF) [file pgen.1011900.s040.tif]

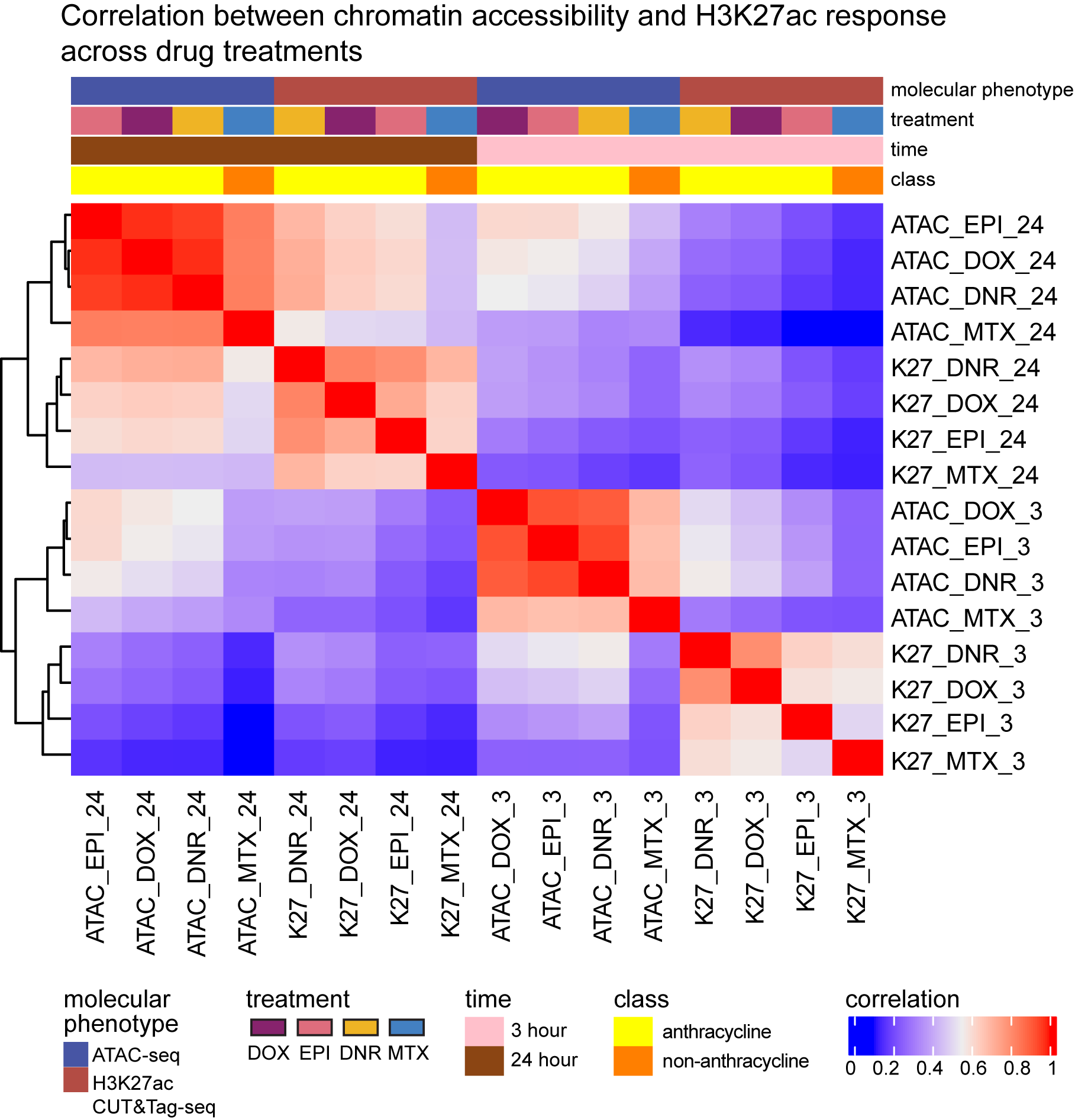

Supplement: S17 Fig — Pearson correlation of log2 fold change of drug response in regions shared between ATAC-seq and H3K27ac CUT&Tag data. Shared regions are defined as regions which overlap by at least 1 bp. A total of 19,894 (98.8%) H3K27ac regions overlap open chromatin regions. Top bars are colored by molecular phenotype (ATAC-seq: dark blue; H3K27ac CUT&Tag: maroon), treatment (DOX: mauve; EPI: pink; DNR: yellow; MTX: blue), time (three hours: pink; 24 hours: brown), and class (anthracycline: yellow; non-anthracycline: orange). (TIF) [file pgen.1011900.s041.tif]

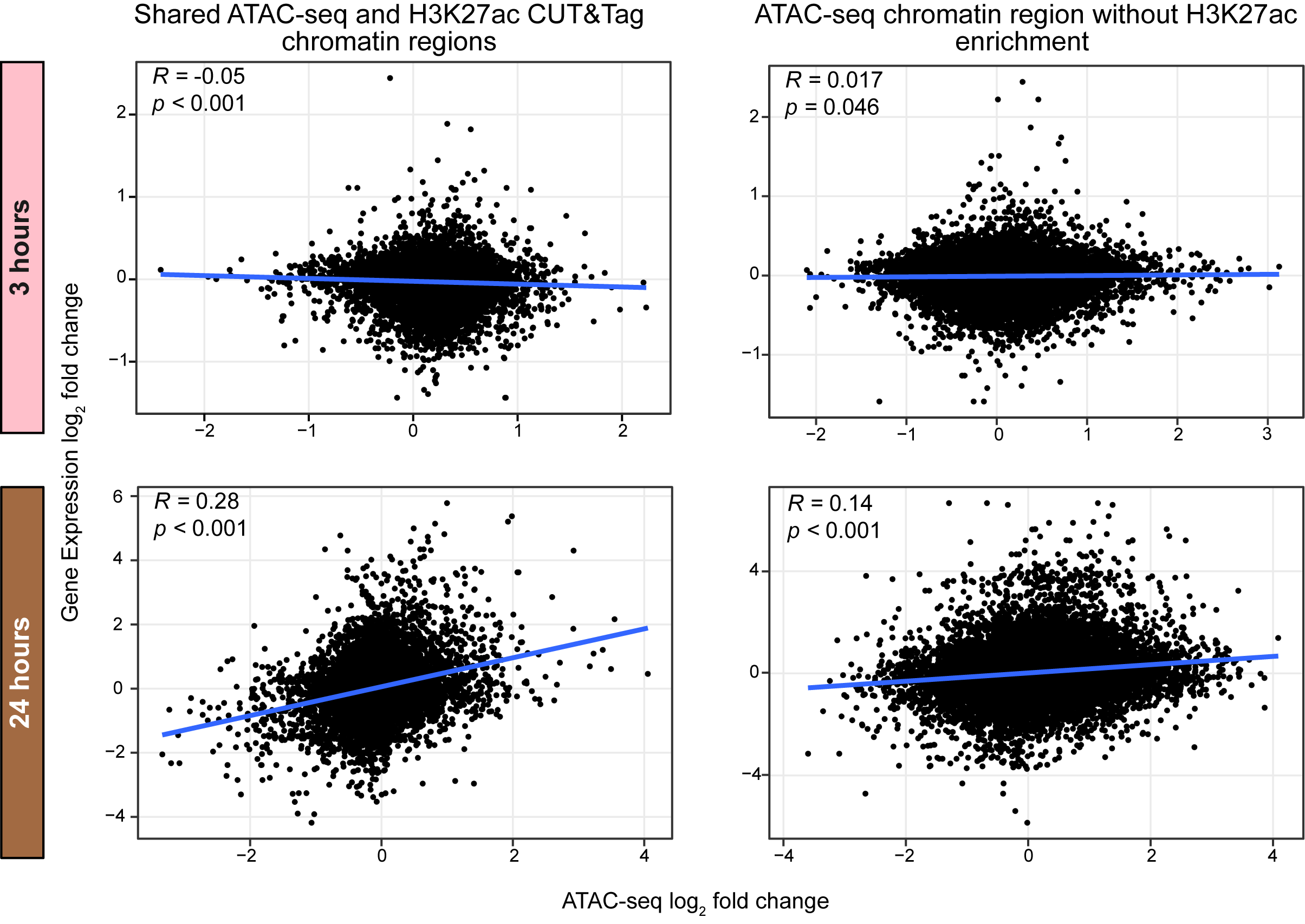

Supplement: S18 Fig — Correlation between drug response of open chromatin regions shared with H3K27ac regions and drug response of nearby genes, and correlation between drug response of open chromatin regions that do not overlap H3K27ac regions and drug response of nearby genes. Only open chromatin regions that are within +/- 2 kb of an expressed gene TSS [16] are shown (n = 10, 859). (TIF) [file pgen.1011900.s042.tif]

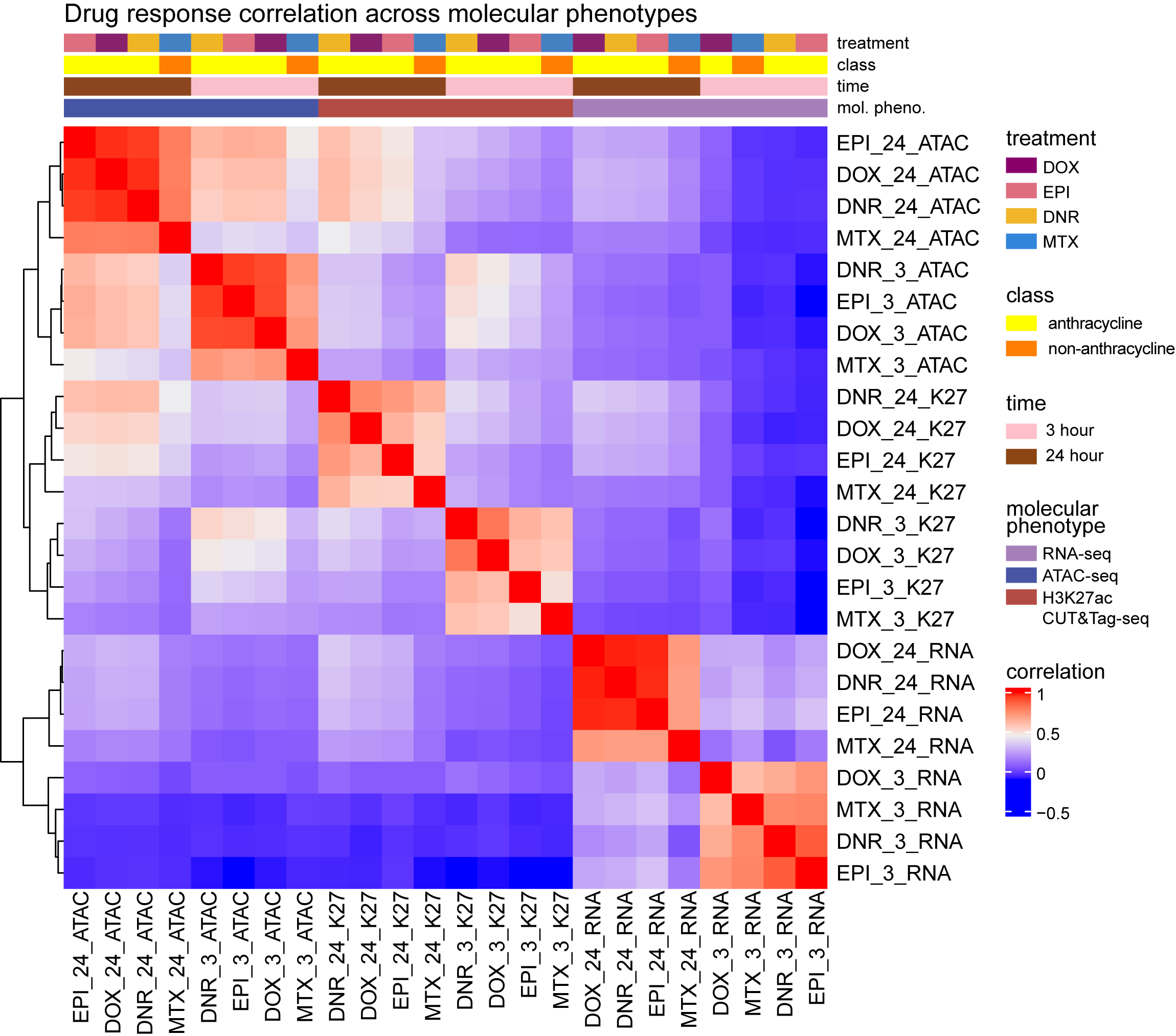

Supplement: S19 Fig — 10,859 open chromatin regions with H3K27ac enrichment were associated with the nearest expressed gene within 2 kb of the TSS [16]. A Pearson correlation of the log2 fold change in response to each drug was performed for three molecular phenotypes (gene expression, chromatin accessibility and H3K27ac enrichment). Top bars are colored by molecular phenotype (RNA-seq: purple; ATAC-seq: dark blue; H3K27ac CUT&Tag: maroon), treatment (DOX: mauve; EPI: pink; DNR: yellow; MTX: blue), and time (three hours: pink; 24 hours: brown). (TIF) [file pgen.1011900.s043.tif]

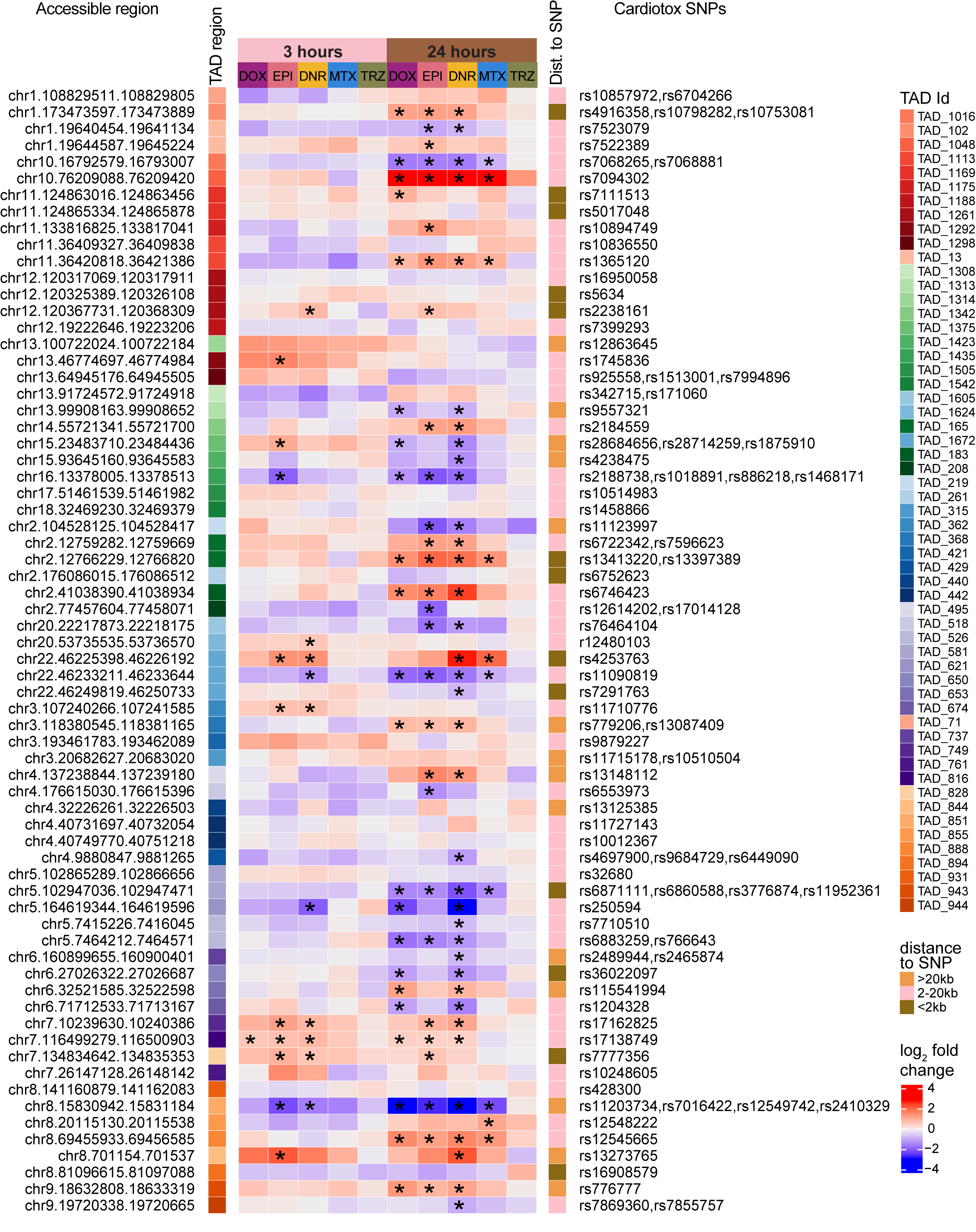

Supplement: S20 Fig — Drug response effect size (log2 fold change) of accessible regions within the same TAD as cardiotoxicity SNPs. Asterisk represents regions that are classified as DARs in each treatment. (TIF) [file pgen.1011900.s044.tif]

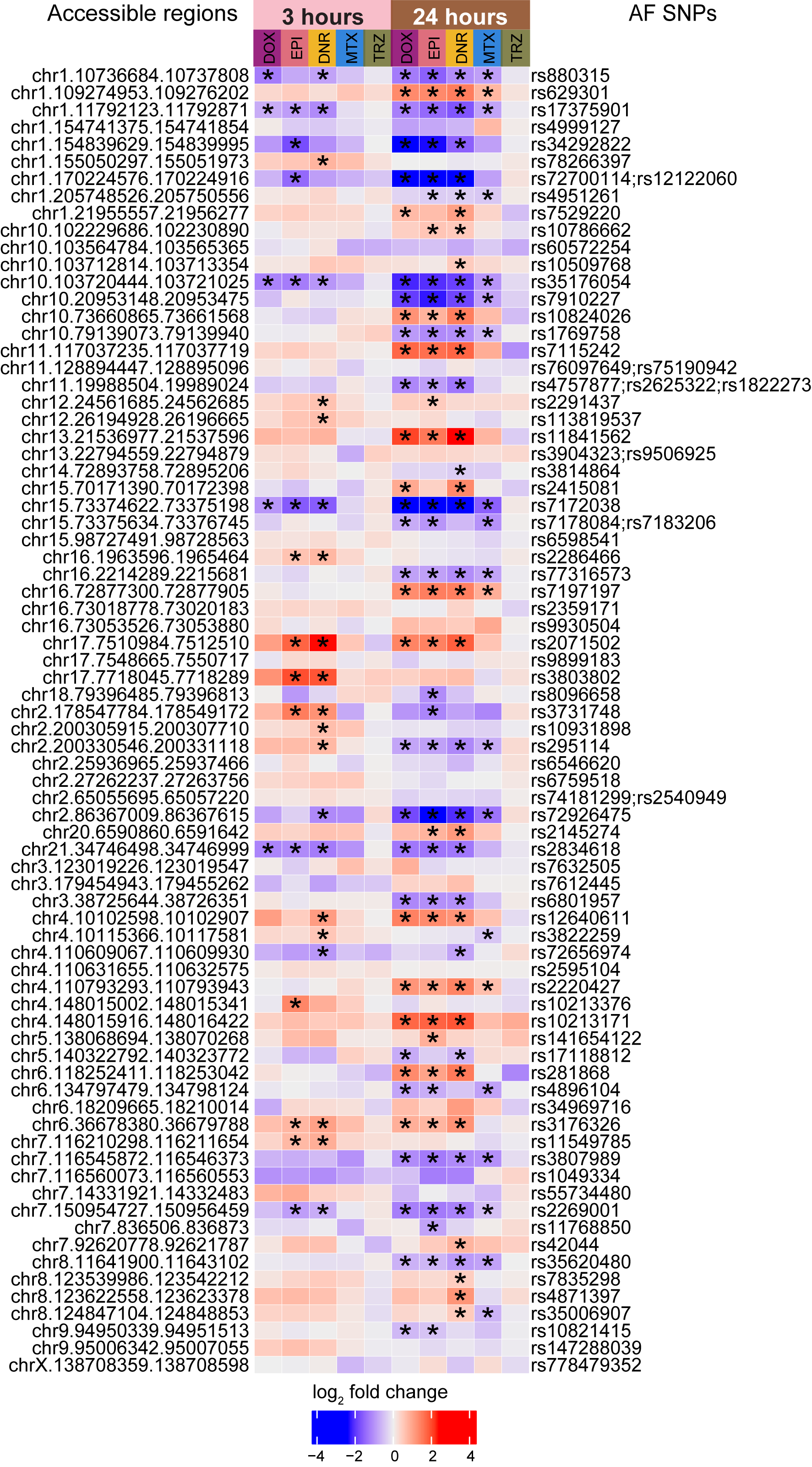

Supplement: S21 Fig — Drug response effect size (log2 fold change) of accessible regions overlapping AF SNPs. Asterisk represents regions that are classified as DARs in each treatment. (TIF) [file pgen.1011900.s045.tif]

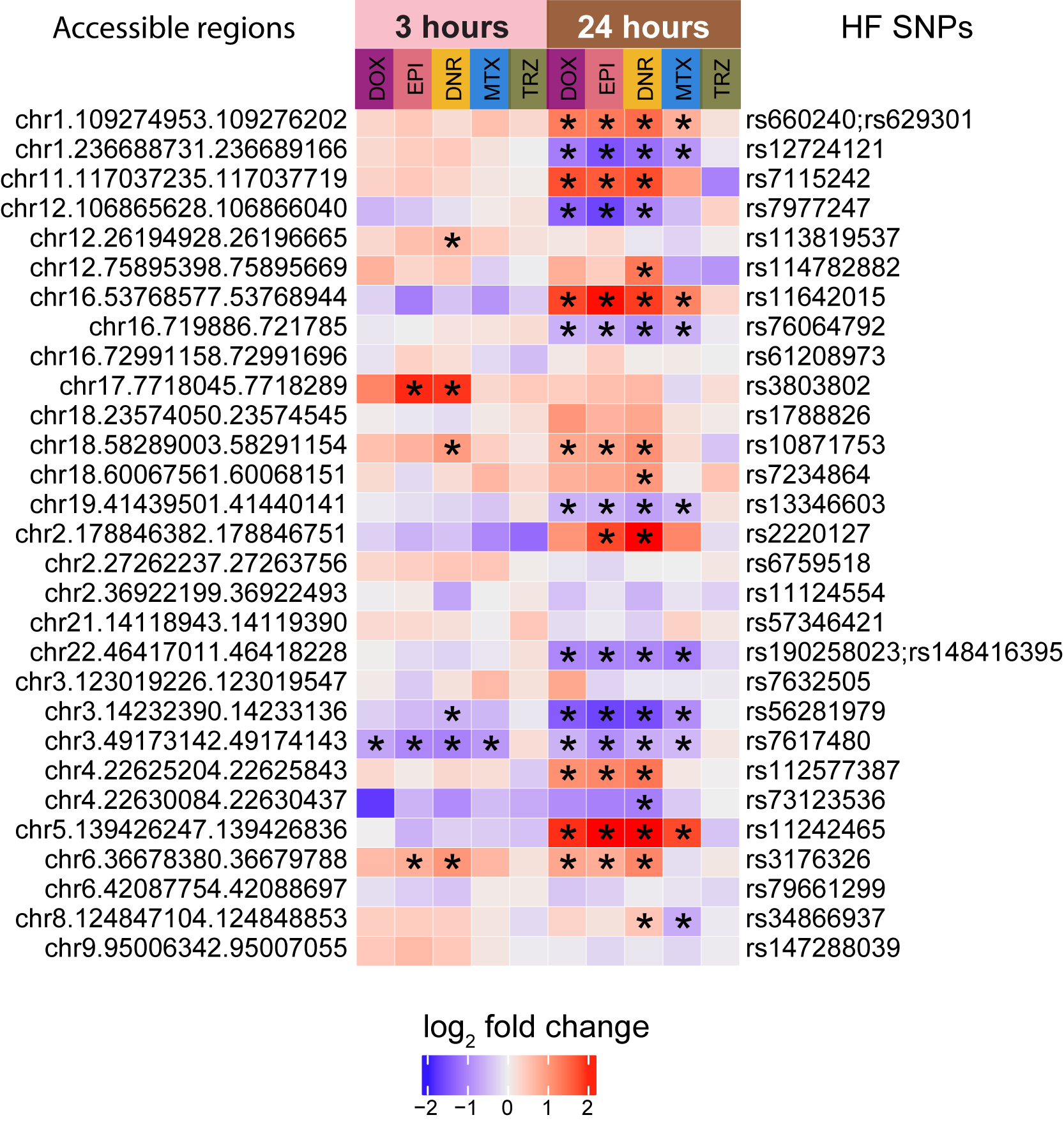

Supplement: S22 Fig — Drug response effect size (log2 fold change) of accessible regions overlapping HF SNPs. Asterisk represents regions that are classified as DARs in each treatment. (TIF) [file pgen.1011900.s046.tif]
