# Supplementary material for: Anthracyclines induce global changes in cardiomyocyte chromatin accessibility that overlap with cardiovascular disease loci
Source: PLoS Genet. 2025 Oct 14;21(10):e1011900. doi: 10.1371/journal.pgen.1011900 (PMC12561927; doi:10.1371/journal.pgen.1011900)
Supplement: Attachment — Submitted filename: Matthews_reviewer_comments_250802.pdf [file pgen.1011900.s047.pdf]

Dear Editor,

Thank you for sending the reviews. We have addressed the reviewers' comments as detailed below. We believe that these changes strengthen the manuscript. In particular we have:

(i) generated new cellular phenotype data indicating the molecular consequence of anthracycline drugs across individuals and time,
(ii) generated ChIP-seq data for TOP2B, the target of anthracycline drugs, to gain insight into the mechanism behind accessibility changes,
(iii) focused the manuscript on differentially accessible regions (DARs) following each drug treatment instead of a bulk analysis across drugs, which indicates reproducibility and specificity of anthracycline effects,
(iv) determined the effect of chromatin accessibility changes on gene expression,
(v) identified enrichment of atrial fibrillation associated SNPs in DARs and determined that DARs are closer to anthracycline-induced cardiotoxicity SNPs than nonDARs within the same TAD.

During the revision process we noticed an error in our code for identifying DARs which did not account for the individual effect. The result of the error was an increase in the number of regions as follows: 3 hour DOX (514 to 3,473), 3 hour DNR (8,912 to 22,738), 3 hour EPI (4,081 to 14,234), 3 hour MTX (44 to 804), 3 hour TRZ (0 to 1), 24 hour DOX (47,624 to 64,820), 24 hour DNR (63,209 to 79,995), 24 hour EPI (48,475 to 66,501), 24 hour MTX (5,830 to 24,250). While these changes impacted exact enrichment numbers for downstream analyses, the overall results are unchanged.

We believe that these changes improve the manuscript and hope that you will find our paper suitable for publication.

As per journal requirements, we have provided both a clean version of the revised manuscript as well as one with track changes. The line numbers in the responses below refer to the version with track changes.

Reviewer #1:

Major comments:

1. While breast cancer patients are treated for long time with Anthracyclines, the current study evaluates their effect on iPSCs derived heart cells for 3 and 24 hrs. It is not clear why was this time points chosen. Even with 3 and 24hrs, there is stark differences in chromatin accessibility profiles, indicating a different treatment period will have more chromatin changes, thus it is difficult to infer any valid conclusions from these datasets.

Cardiotoxicity from breast cancer drugs can develop immediately, after a single dose, or years after treatment (Cardinale, Circulation, 2015). There is therefore a need to understand both short- and long-term effects. We agree that this was not clear in the manuscript.

These specific timepoints were chosen given our previous work using these concentrations of drugs that showed extensive changes in gene expression at 24 hours but minimal changes at three hours, as well as effects on cardiac contraction within 24 hours. This study aimed to understand the basis of these gene expression changes at those time points. We agree that this was not clear in the manuscript.

To further justify the timepoints chosen, we tested the effects of the drugs on DNA damage, the proposed mechanism of anthracyclines, at these timepoints across the four individuals. We observed an increase in DNA damage within three hours of treatment indicating the impact of the drugs on the cells.

We have added text to the introduction to indicate that short- and long-term effects of cancer treatments on the heart in lines 156-157. We now justify the choice of timepoints based on our previous data in lines 182-186. Data showing the expression of the DNA damage marker gamma H2AX by immunofluorescence is presented in Fig 1C-D and is described in lines 186-194.

2. Since anthracyclines only affect myocardial function including causing heart failure and arrythmias and do not affect ischemic heart diseases, can the authors do similar experiments in endothelial cells and show that they do not affect endothelial cell chromatin accessibility?

We expect that DOX will affect chromatin accessibility that is both cell type-specific and shared across cell types. Data collection for differentiating iPSC-CMs from multiple individuals, treating them and generating ATAC-seq data took approximately one year. It would not be feasible to repeat the experiment in a new cell type in the requested revision timeline. However, we now include GWAS data for ischemic heart disease and coronary artery disease and show that there is enrichment for atrial fibrillation SNPs, but not ischemic heart disease or coronary artery disease SNPs, in DARs across anthracyclines. We also note that we include Trastuzumab and show that drug-induced chromatin changes, including those at CVD risk loci, are specific to TOP2 inhibitors.

Data for the ischemic heart disease variant enrichment is displayed in Fig 6A and described in lines 688-697. A quantitative assessment of drug-induced chromatin changes at atrial fibrillation GWAS variants is shown in Fig 6B and described in lines 697-700.

3. Mature human cardiomyocytes are bi-nucleated. It appears that the authors used iPSC derived immature cardiomyocytes. Please provide data to show that the cardiomyocytes used here are mature cardiomyocytes that mimics patient conditions.

Our differentiation protocol includes two steps to mature the iPSC-CMs. First, while cells beat and express cardiac troponin T within seven days of the start of the differentiation, we maintain iPSC-CMs until Day 27 as time in culture associates with a more mature state. Second, we culture our cardiomyocytes in galactose-containing media which more closely mimics the metabolic conditions of adult cardiomyocytes than glucose-containing media. This protocol has been used in a number of studies (Ward, eLife, 2021; Banovich, Genome Research, 2018; Matthews, PLOS Genetics, 2024; Johnson, iScience, 2025) that have indicated concordance in the regulome, transcriptome and proteome between our iPSC-CMs and heart tissue. We now include immunofluorescence images of alpha actinin and cardiac troponin T staining of the iPSC-CMs from each of the four individuals. These images show the sarcomeric organization and structure of our cells, indicative of their level of maturity. Nevertheless, we agree that immaturity is a common feature of iPSC-derived cell types.

We now include images of our iPSC-CMs derived from each individual in Fig 1B which is described in lines 179-180. We also state that maturity is a limitation in the discussion in lines 1039-1042.

4. Does anthracycline treated human iPSC cardiomyocytes show arrhythmia and heart failure phenotype? In absence of these phenotypic observations, chromatin accessibility changes,

although informative, do not contribute much insight into drug induced cardiotoxicity. The authors should provide electrophysiology data of the iPSC cardiomyocytes after anthracycline treatment to justify the timing and concentration of the drugs used.

In our earlier study, using iPSC-CMs treated with these drugs at the same concentrations and timepoints, we measured several aspects of calcium signaling including peak amplitude, rising and decay slopes, which provides inference about drug effects on contraction. These results show that at these concentrations of drugs, various aspects of calcium signaling are affected within 24 hours.

We now explicitly state these results when justifying the chosen treatment concentrations and timepoints in lines 182-186.

5. In absence of HiC data, associating accessibility regions and cardiovascular diseases risk loci within 20 kb is bit of a stretch. Can the authors perform HiC to prove that these accessibility regions and the CVD risk loci are in the same loop or TAD.

We obtained TAD regions identified in human heart tissue (Wang, Genome Biol, 2018; Leung, Nature, 2015). We now consider all accessible regions and SNPs within the same TAD rather than a distance-based approach. We find that within a TAD, anthracycline DARs are closer to cardiotoxicity SNPs than nonDARs. We also highlight the three cardiotoxicity-associated SNPs that overlap DARs. For CVD risk loci in Fig 6 we consider only direct overlaps between accessible regions and SNPs.

We include these results in Fig 5A-C, which are described in lines 658-686.

6. Overall, the molecular basis of anthracycline induced gene expression and cardiotoxicity is not clear from this study.

We agree that the connection between chromatin changes and gene expression changes should be further explored. We therefore performed several new analyses. In response to a comment from Reviewer 2 about the relevance of the response motifs, we now focus on DARs for each drug and timepoint. This makes the integration with the RNA-seq data clearer. First, we show that 24-hour DARs for DOX, EPI, DNR and MTX are enriched for Differentially Expressed Genes (DEGs) compared to regions that are not DARs. At the three-hour timepoint, only DNR DARs are enriched for DEGs likely due to the fact that there are many more DARs in response to DNR than the other drugs at this timepoint. We then focused our gene ontology analysis on DEGs that are close to DARs. We find processes related to mismatch repair amongst all anthracyclines but not MTX. DNR induces unique processes including glycerophospholipid metabolism. MTX treatment induces changes in the cell cycle and p53 signaling. We also investigated which TF motifs are within DARs proximal to DEGs. We find the FOS:JUNB transcription factor motifs are enriched amongst the anthracyclines but not MTX. MTX is enriched for KLF15 and NFYA amongst others.

Anthracyclines target TOP2, and the TOP2 beta isoform highly expressed in the heart is thought to mediate the cardiotoxic effects on the heart. To gain further insight into the mechanism behind chromatin accessibility changes in response to anthracyclines, we generated ChIP-seq data for TOP2B in cardiomyocytes. We find that 95% of the 5,410 TOP2B peaks localize within accessible chromatin. TOP2B binding sites are enriched amongst three-hour DARs across drugs but are depleted at 24 hour DARs indicating that early changes in chromatin accessibility are associated with binding of the target of anthracyclines.

We include the overlap with DAR-DEGs in Fig 4C, which is described in lines 619-625. We include the gene ontology analysis of the DAR-DEG pairs in Fig 4E, which is described in lines 639-644. The transcription factor motif analysis is presented in Fig 4D and described in lines 627-637. We include TOP2B data in Fig 3A-E, Fig 5C and Fig 6C which is described in lines 280-344, lines 681-682, line 741-742.

Minor:

1. Line 33: it appears that iPSC cardiomyocytes were derived from four individuals treated with anthracyclines: please revise to reflect that iPSC cardiomyocytes derived from four healthy individuals were treated with anthracyclines to evaluate their role on chromatin accessibilities.

This is indeed more accurate. We have made the change on line 33.

2. Line 167: left ventricle?

This is indeed more accurate. We have made the change now on line 216.

3. Line 201: Is it log10 fold change or log2 fold change?

This should be log2 fold change. We have made the change now on line 252.

Reviewer #2:

Major:

1. I am somewhat confused by how the data is presented. Some of the data is presented in aggregate as a box plot by condition, which shows the bulk variation among individuals. This conveys the message that in general, individuals respond in a similar way, and that the message is that most of the treatments cause a similar pattern of responses at the level of accessible chromatin. I believe that this may be the intended message. However, selected browser tracks show each of the conditions per individual, showing the response to treatment in each individual. This presentation conveys that a given individual may respond differently at the level of chromatin accessibility to the various treatments. Unfortunately, this mix of presentation occurs throughout the manuscript.

This is indeed confusing. Our intention, with this small number of individuals, is to show the effects of drug treatment that occur beyond inter-individual variation as mentioned. We have no individual-specific analysis. Our experimental batches correspond to individuals and so comparison between them would be challenging.

We have replaced all genome browser tracks from a single representative individual with tracks that have aggregated data across all individuals. This affects Fig 2F, Fig 3A, Fig 5C, Fig 6C.

2. Further, the data is presented in aggregate based on time point and treatment. Many correlation plots are presented for all of the conditions. However, I don't have a good sense about how similar the samples are within a group (ie. vehicle by individuals A-D) at the level individual accessible chromatin regions, except for some selected browser tracks. I think that this could be accomplished by a heatmap of statistically-robust differentially accessible peaks. (Apologies if it's

presented, and I have missed it). This information would be important to convey the variation within each condition by individual (ie. showing Vehicle for individuals A-D) for then comparing with treatment conditions (ie. Treatment X for individuals A-D). If I understand correctly, this would align with how the data was analyzed for treatment-vehicle comparisons, although this is obscured in how the data is currently presented.

Indeed, all drug treatment effects were tested relative to Vehicle. We have now considered all Differentially Accessible Regions (DARs) per drug and timepoint and performed hierarchical clustering on the accessibility levels (log_2 cpm) in these regions across drug and vehicle. As expected, samples cluster based on whether they were treated with drug or vehicle, and the level of variability in these regions is now evident.

We include heatmaps of each set of DARs in Fig S10. The data is described in lines 247-250.

3. I can appreciate the effort to stratify the accessibility responses using the joint Bayesian into shared patterns. However, it is not clear to me if relevant biological insights have been derived from this.

We agree with this comment. We now focus the manuscript on DARs for each drug which is easier to interpret and shows the reproducibility of our findings across ACs and highlights the differences with MTX.

Much of the manuscript is rewritten to focus on this perspective. We show an in-depth analysis of the set of DARs in Fig 2, a characterization of these DARs in Fig 3, and use these regions to connect to RNA-seq data in Fig 4.

4. I understand that many of the acronyms correspond to those in the authors' previous 2024 publication. However, the number of acronyms, especially in Figure 1, borders on overwhelming, especially as many acronyms, if not most, are not common.

We agree. As mentioned in response to Comment 3. We now focus on DARs and not cormotif motifs and their sub-categories.

5. Although a lot of data is presented, it's not clear to me the relevance of Fig 1-4, as presented.

We agree. We have restructured and re-focused this analysis based on DARs, and the connection to gene expression. Relevant changes are described in response to Reviewer 1 Comment 6.

6. I see p-values for some of the data in the manuscript, but it is not apparent to me for others. (Apologies if I have missed this). Please also consider adding p-values in the Figures or the figure legends. For example, For Fig S4, is the overlap with the in vivo heart sample statistically significant? For Fig 6 and 7, are SNPs significantly enriched in differentially accessible chromatin regions? Same with the comparisons in B, C, and D for Fig 6 or C, D, and E for Fig 7. Are SNPs from heart failure or atrial fibrillation are statistically enriched in differentially accessible regions by cancer therapies in iPSC-derived cardiomyocytes? If yes, please show the relevant adj p-value by statistical metric.

The overlap between our ATAC-seq peaks and heart tissue ATAC-seq peaks is significant based on permutation analysis.

Regions or genes that are represented in boxplots are a DAR or DEG in response to at least one drug at at least one timepoint. We agree this should have been indicated in the plots.

For the DOX cardiotoxicity SNP analysis, we now test whether there is a difference between the distance between SNPs and DARs and nonDARs in the same TAD. We find DARs are closer to cardiotoxicity-associated SNPs.

For the CVD SNP analysis we tested whether CVD SNPs are more likely to overlap a DAR than a nonDAR across drugs. We find that atrial fibrillation SNPs are enriched across anthracycline DARs at three hours but not SNPs for other cardiovascular diseases. We also test the difference in the response to TOP2 drugs and trastuzumab at atrial fibrillation-associated variants and show that there is an increased response in TOP2 drugs.

We add the significance related to Fig S4B on line 218. We indicate DARs with an asterisk in boxplots in Fig 3E, Fig 5D, Fig 5E, Fig 6D, Fig 6E, Fig 6F, Fig S11. We include the cardiotoxicity SNP distance analysis in Fig 5 and refocus this figure to show this data and a TAD containing cardiotoxicity SNPs that are close to DARs. This analysis is described in lines 659-664. The CVD enrichment analysis is represented in Fig 6A and described in lines 693-697. The drug response comparison at atrial fibrillation-associated loci is represented in Fig 6B and described in lines 697-699.

Minor:

1. I assume the authors refer “heart eSNP” as a SNP in a heart enhancer, although I could be mistaken. This ambiguity could be clarified for the reader with a definition and a reference to the publication that defines this set of eSNPs.

eSNP refers to a SNP that is an expression quantitative trait locus (eQTL). We used eSNP to denote the SNP and eGene to denote the gene whose expression is associated with the SNP. We appreciate that this naming convention may not be clear to a broad audience. We now refer to the association between the gene and the SNP as an eQTL and the gene as eGene and no longer use eSNP.

We refer to eQTLs in Fig 5B, Fig 6C and eGenes in Fig 5E and Fig 6F and define this in line 676.
