# Supplementary material for: Anthracyclines induce global changes in cardiomyocyte chromatin accessibility that overlap with cardiovascular disease loci
Source: PLoS Genet. 2025 Oct 14;21(10):e1011900. doi: 10.1371/journal.pgen.1011900 (PMC12561927; doi:10.1371/journal.pgen.1011900)
Supplement: Attachment — Submitted filename: Matthews_Reviewer_response_250914.pdf [file pgen.1011900.s048.pdf]

Dear Editor,

Thank you for sending the reviews. I believe we have addressed all comments with edits to the manuscript text and figures as outlined below. Line numbers refer to the manuscript with track changes.

Reviewer #1:
This article provides simple association of disease risk SNPs and treatment induced chromatin accessibility changes, which should be of broad interest, however, it doesn't go beyond this to provide applications or implications of this study, which the authors should discuss more.

Personalized medicine promises to consider treatment options that best suit individual patients based on their individual genotypes and phenotypes. Genotyping of individuals to assess risk for disease or adverse drug events is becoming increasingly available. However, there is still a gap determining which SNPs are relevant to a phenotype. Our study identifies and provides thousands of genomic locations that robustly associate with treatment of a panel of ACs in cardiomyocytes. While this study alone is not able to predict CVD risk in specific women being treated for breast cancer, the data generated can serve as a functional annotation following genotyping of individuals in a cancer patient population to stratify risk and inform on treatment schedules and early disease screening.

We agree that the implications and applications of our work were not clear. We have: 1) added a paragraph to the discussion that provides applications and implications of the study in lines 553-561, 2) expanded on our summary paragraph in lines 583-596 and 3) added to our abstract in lines 48-50. These three paragraphs are also included below:

'Personalized medicine promises to consider treatment options that best suit individual patients based on their individual genotypes and phenotypes. Genotyping of individuals to assess risk for disease or adverse drug events is becoming increasingly available. However, there is still a gap determining which SNPs are relevant to a phenotype. Our study identifies and provides thousands of genomic locations that robustly associate with treatment of a panel of ACs in cardiomyocytes. While this study alone is not able to predict CVD risk in specific women being treated for breast cancer, the data generated can contribute to functional annotation following genotyping of individuals in a cancer patient population to stratify risk, and inform on treatment schedules and early disease screening.'

'While many studies have demonstrated that DOX induces gene expression changes in cardiomyocytes that are relevant for CVD (13,15,16), the effects of ACs on chromatin, a genomic feature that can be directly associated with CVD SNPs, were unknown. We generated global chromatin accessibility data in iPSC-CMs from four individuals treated with four TOP2i drugs and TRZ, at two timepoints, to determine the impact of these drugs on chromatin. Our data supports the current understanding that ACs damage cardiomyocytes through TOP2B inhibition and the generation of DNA double-strand breaks. We uncovered strong effects of ACs on chromatin accessibility that associate with gene expression changes, and identified CVD-associated variants that localize in AC-responsive chromatin regions. These results suggest that ACs remodel chromatin in cardiomyocytes allowing for the direct functional annotation of genetic variants in individuals being treated with these drugs rather than indirect measurements of linked gene expression. We believe that this data from the iPSCORE panel of genotyped individuals will also provide a resource for determining the contribution of AC treatment to risk of developing CVD.'

'The identified drug-responsive chromatin regions can be used to annotate variants in cancer patient populations to contribute to risk estimation for CVD.'

Minor points

1. Fig1b: authors should high quality images of show single cardiomyocytes

We have replaced the images of cardiomyocytes in Fig 1B with ones that indicate the morphology of single cells.

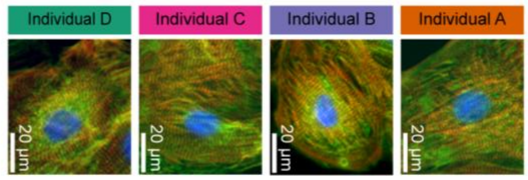

2. Table S1 shows >98% cardiomyocytes, but authors should show a FACS plot showing the purity of cardiomyocytes

We have added the raw FACS data in Fig S1. We include both the TNNT2 density distribution (Fig S1A) and the purity reported based on our gating criteria (Fig S1B). The figure is referenced in lines 144-145 and described in the legend in lines 1534-1538.

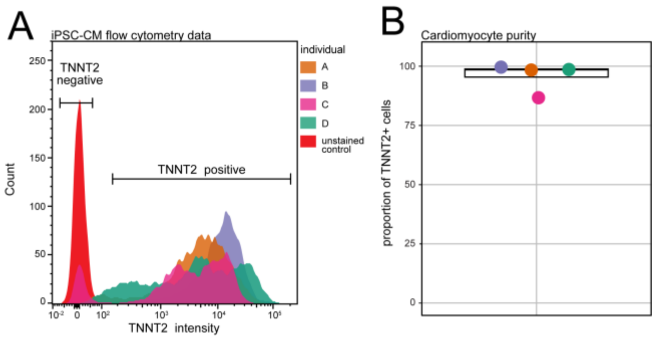

3. Fig 3a, no input sequence shown or peak called shown making it difficult to understand if the ChIPseq signal is real or background

This indeed should have been included. We have added an Input track and a peak track to Fig 3A. The figure is described in the legend in lines 1455-1458.

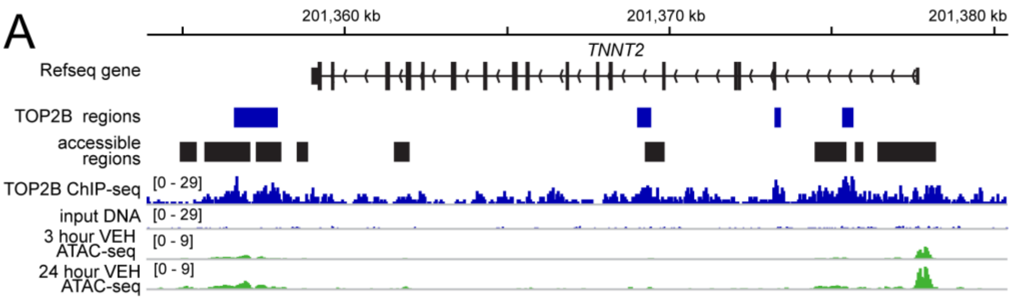

4. Line 244: Fig 3A, should be Fig 3F

Thank you, yes this was an error. This line (now line 259) now references Fig 3F.

Reviewer #2:

Minor comments:

1) I think that the comparison of DARs to CARs is useful in many contexts of the manuscript, especially when showing significance in DARs but not CARs. However, I'm not aware of other publications that supports the notion that the proximity of a SNP to DARs compared to CARs is meaningful, especially when chromatin is often considered in a 3-D context. Please include support for this if available in the literature. Otherwise, I think that the few mentions of the comparison of the distance of SNP to DARs and CARs (and the accompanying figure panel) of this could be amended without substantially weakening the manuscript.

GWAS studies typically test a locus by tagging a SNP in the region and genotyping participants at this SNP. There are likely to be many SNPs in linkage disequilibrium (LD) with these lead variants in the locus that are not typed. Identifying the SNP that is causal to a phenotype is therefore challenging. Measures of LD between SNPs decrease with increasing genetic distance given meiotic recombination (eg Bosch, BMC Genomics, 2009: PMID: 19638193). We therefore reasoned that DARs could overlap SNPs in LD with the lead SNP and that this would track with distance from the SNP. It has also been shown that many disease-associated SNPs overlap, or are in LD with SNPs that overlap DHS sites, suggesting that local context beyond the tag SNP is relevant for disease (Maurano, Science, 2013: PMID: 22955828).

While we agree that disease-associated SNPs that directly overlap DARs are more interpretable, we felt that this small dataset of AC-induced cardiotoxicity was worth including given its direct relevance to our study. Given this rationale and the feedback from Reviewer 1 we have retained this analysis in the manuscript.

We now explain the rationale for this analysis in line 376-379 and add to the discussion in lines 520-524 as outlined below.

'We reasoned that SNPs linked to the reported cardiotoxicity-associated SNP in the same genomic region may also be relevant given that many disease-associated SNPs are in linkage disequilibrium with SNPs in open chromatin regions (24) and that linkage disequilibrium between SNPs decays with genetic distance.'

'*We found that in SNP-containing TAD chromatin regions, DARs are closer to these SNPs than CARs suggesting drug-responsive regulatory activity in these regions. This is highlighted in a locus near the PRDX6 gene that shows chromatin accessibility changes in response to AC treatments close to cardiotoxicity-associated SNPs, that also associates with the expression of the DARS2 gene in heart tissue.'*

2) I understood that SNPs of ischemic heart disease and coronary artery disease are used to contrast to SNPs of heart failure and atrial fibrillation. If this is the intention, then the way that line 690 is written makes this point a little confusing.

Yes, this was the intention, but it was not clear in the text. We have edited the text accordingly in line 403.

'CTRD has a range of clinical definitions, therefore we considered two types of CVD that individuals receiving ACs are at increased risk for: heart failure (HF) and atrial fibrillation (AF) as well as ***two unrelated CVD****: ischemic heart disease (IHD) and coronary artery disease (CAD).'*

3) In the paragraph for lines 786-801, I was a little unclear what sentences related to the current work versus discussion of previous publications. For example, line 793-794 is not clear to me what the authors are trying to say.

After re-evaluating this section, we decided that lines 793-794 relating to DOX effects on cohesin binding is not necessary for the message of the paper. We have therefore removed this sentence. We have made minor edits to clarify which work corresponds to our study and which corresponds to work from other studies. The revised paragraph is in lines 453-463.

'The chromatin changes that we observe in response to ACs in iPSC-CMs are in line with the changes observed in cancer cells. DOX treatment of osteosarcoma cell lines induced ~20,000 chromatin accessibility changes with concomitant changes in chromatin organization and condensation (31). The authors of this study showed that etoposide, an unrelated TOP2i, had minimal effects on chromatin accessibility. We find that MTX, another unrelated TOP2i, has fewer effects on chromatin accessibility compared to ACs. Thousands of chromatin accessibility changes also exist between breast cancer cell lines that are sensitive to DOX treatment compared to those that are resistant thereby providing further support for DOX effects on chromatin (21). Interestingly, hypoxia and oxidative stress in iPSC-CMs induce minimal effects on chromatin accessibility across 15 individuals despite thousands of gene expression changes, which suggests that stress does not uniformly affect chromatin accessibility in cardiomyocytes (32).'

4) Line 877: unless I missed it, I did not see occupancy data for JUN/FOS or p53. I would suggest slight modification of "binding" to something like "accessibility of motifs"…

Yes, we did not measure binding and therefore the statement is misleading. We have edited now line 500 accordingly.

'Our results suggest that chromatin changes are associated with ***accessibility of the motif*** *for this stress-responsive transcription factor.'*
